# The *mex-3* 3′ untranslated region is essential for reproduction during temperature stress

**DOI:** 10.1242/dev.204740

**Published:** 2025-09-11

**Authors:** Hannah E. Brown, Haik V. Varderesian, Oscar Lam, Sara A. Keane, Sean P. Ryder

**Affiliations:** Department of Biochemistry and Molecular Biotechnology, University of Massachusetts Chan Medical School, Worcester, MA 01605, USA

**Keywords:** RNA-binding protein, Untranslated region, Nematode, Germline, Reproduction, Maternal RNA

## Abstract

Organisms must sense temperature and modify their physiology to survive environmental stress. Elevated temperature reduces fertility in most sexually reproducing organisms. Maternally supplied mRNAs are required for embryogenesis. They encode proteins that govern early embryonic patterning. RNA-binding proteins are major effectors of maternal mRNA regulation. MEX-3 is a conserved RNA-binding protein essential for anterior patterning of *Caenorhabditis elegans* embryos. We previously demonstrated that the *mex-3* 3′ untranslated region (3′UTR) represses MEX-3 abundance in the germline yet is mostly dispensable for fertility. Here, we show that the 3′UTR is essential during thermal stress. Deletion of the 3′UTR causes a highly penetrant, temperature-sensitive embryonic lethality phenotype distinct from the *mex-3* null phenotype. Loss of the 3′UTR decreases MEX-3 abundance specifically in maturing oocytes and early embryos during temperature stress. Dysregulation of *mex-3* reprograms the thermal stress response by reducing the expression of hundreds of heat-shock genes. We propose that a major function of the *mex-3* 3′UTR is to buffer MEX-3 expression during fluctuating temperature, ensuring the robustness of oocyte maturation and embryogenesis.

## Teaser

**Highlighted Article:** Deletion of the *mex-3* 3′UTR in *C. elegans* embryos reveals that the 3′UTR is essential for reproductive robustness during temperature stress and that dysregulation of MEX-3 alters the heat-shock response.

## INTRODUCTION

Thermoregulation is crucial for sexual reproduction throughout the plant and animal kingdoms (Abram et al., 2017; Hansen, 2009; Walsh et al., 2019). Mammals evolved external testicles to ensure that spermatogenesis occurs below body temperature (Lovegrove, 2014). In chickens and mice, short exposure to thermal stress results in prolonged reduction of sperm concentration (Walsh et al., 2019). In cows, ovulation and implantation are compromised in warmer seasons (Hansen, 2009). In humans, temperature stress can impact gestational health and have developmental consequences in the children of affected mothers (Dreier et al., 2014).

Ectotherms, which do not thermoregulate, employ a variety of adaptations to ensure robust embryogenesis in thermal stress (Abram et al., 2017). Hornets regulate hive temperature by beating their wings at the nest entrance to ensure adequate ventilation (Riabinin et al., 2004). Turtle embryos reorient inside of the shell to ensure optimal temperature (Du et al., 2011). In certain reptiles and fish, sex determination is controlled by external temperature cues (Bull and Vogt, 1979; Conover and Heins, 1987). Although it is generally accepted that thermoregulation is fundamental to reproductive success, the molecular mechanisms that protect gametes and embryos from thermal stress are not well understood.

Several aspects of *Caenorhabditis elegans* reproduction are sensitive to temperature. The optimal reproductive temperature of this species is 20°C (Stiernagle, 2006). Prolonged thermal stress above 28°C prevents embryonic development and hatching (Aprison and Ruvinsky, 2014). Thermal stress above 31°C prevents egg production (Aprison and Ruvinsky, 2014). Prolonged exposure to 27°C causes transgenerational sterilization – the progeny of stressed adults are sterile (Begasse et al., 2015). L1 larvae that experience temperature stress enter an alternative life cycle, producing temperature-resistant dauer larvae that can persist for long periods of time before re-entering the path to adulthood when conditions are more favorable (Byerly et al., 1976).

In general, the higher the temperature and the longer the exposure, the more significant the impact to reproductive fecundity. Yet even limited duration exposure to elevated temperature can affect gametogenesis, ovulation rate and hatch rate, depending on time and temperature, with variable recovery rates upon shifting to the optimal environment (Anderson et al., 2011; Aprison and Ruvinsky, 2014; Begasse et al., 2015; Kurhanewicz et al., 2020; Rogers and Phillips, 2020). Short duration exposure to heat alters the sex ratio in *C. elegans*, increasing the number of chromosomal nondisjunction events that occur during spermatogenesis resulting in more XO males (as opposed to XX hermaphrodites) (Byerly et al., 1976). One of the earliest embryonic patterning events, the timing of the first cell division cycle, is sensitive to temperature (Begasse et al., 2015). The rate of the first cellular division scales exponentially with increasing temperature. The underlying mechanisms behind these phenomena are not clear, but the types of defects and their timing during the reproductive cycle suggest an important role for maternal gene products.

In most animals, maternally deposited proteins and mRNAs are required for fertility and embryo development after fertilization (Farley and Ryder, 2008). Stored maternal mRNAs are regulated in *cis* by elements found within their untranslated regions and in *trans* by RNA-binding proteins (RBPs) and microRNAs (miRNAs). In *C. elegans*, the germline undergoes several important transitions, including a switch from mitosis to meiosis, a transition from individual germ cells to a syncytial state, differentiation into either sperm or eggs, and the maturation of oocytes (Albarqi and Ryder, 2022). Following fertilization, embryos undergo transitions that govern body axis formation, segregation of the germline from somatic cells, and cell fate specification prior to zygotic gene activation. Maternal mRNAs and RBPs contribute to each of these transitions in the germline and embryo.

**Fig. 1.**
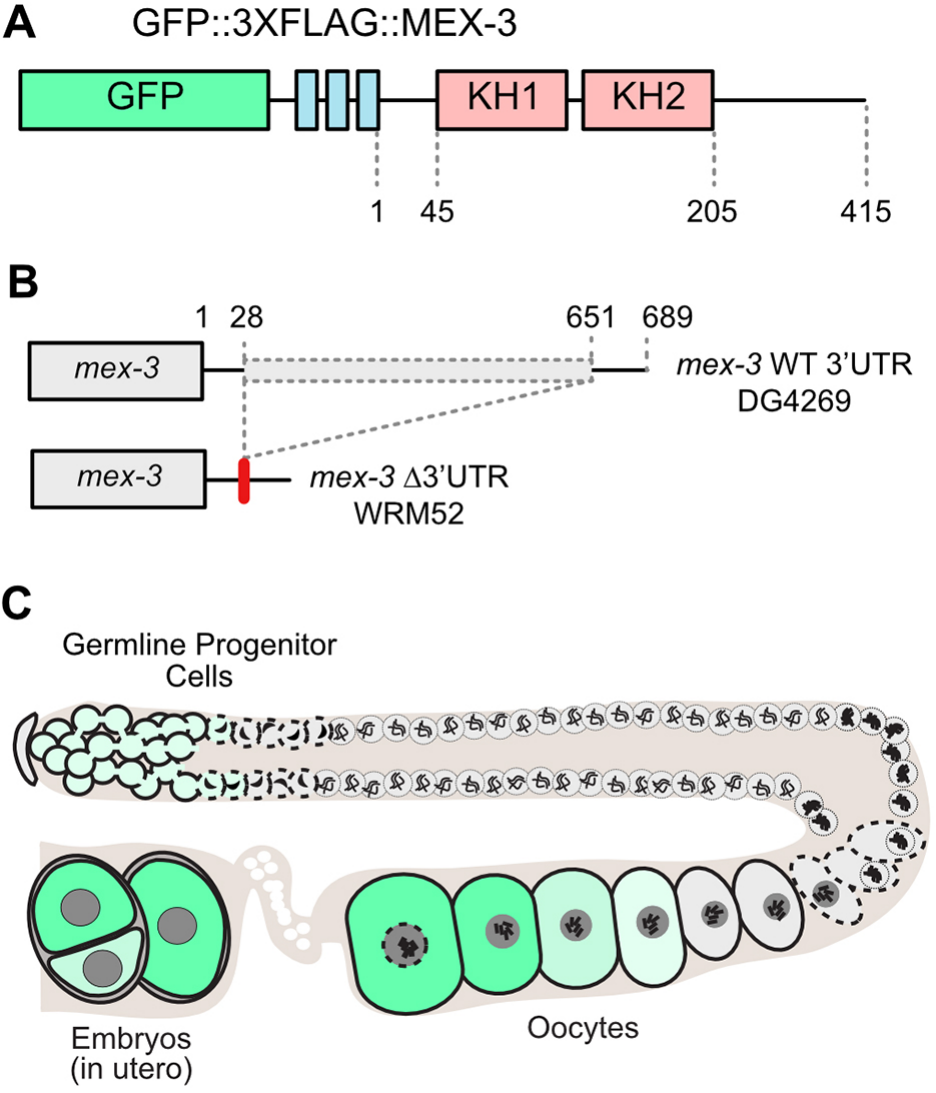
**MEX-3 is a germline RBP required for anterior cell fate specification in the embryo.** (A) The endogenous MEX-3 protein contains two KH domains and was tagged with GFP and 3X FLAG at the N terminus (DG4269) (Tsukamoto et al., 2017). (B) The deletion allele that removes 624 of the 689 nucleotides of the 3′UTR was made in this strain background (WRM52) (Albarqi and Ryder, 2021). (C) Diagram of a hermaphrodite gonad, with MEX-3 expression represented in green and darker colors indicating stronger expression.

MEX-3 is a conserved KH-domain RBP required for anterior cell fate specification in early embryogenesis (Fig. 1) (Draper et al., 1996; Huang et al., 2002). Loss-of-function *mex-3* alleles cause anterior cells to adopt posterior-like fates, leading to excess muscle and germline lineage cells in terminal embryos. In the adult germline, MEX-3 expression is restricted to the distal mitotic region and the proximal oocytes (Draper et al., 1996). In the embryo, expression is enriched in the anterior cells until the 8- to 16-cell stage, when expression is lost. In the posterior cells, the remaining MEX-3 protein is sequestered in P granules in germline progenitor cells (Draper et al., 1996). In animals depleted of sperm, MEX-3 aggregates into large ribonucleoprotein (RNP) granules in oocytes (Elaswad et al., 2022; Jud et al., 2008).

The *mex-3* 3′UTR is sufficient to confer the MEX-3 expression pattern to a reporter transgene in the germline (Kaymak et al., 2016; Merritt et al., 2008). The 3′UTR is also necessary for patterned expression from the endogenous locus (Albarqi and Ryder, 2021). We previously showed that a 624 base pair deletion mutation in the endogenous 3′UTR [mex-3 (spr9[*tn1753]), hereafter referred to as *gfp::3xflag*::*mex-3*::Δ3′UTR] increases MEX-3 abundance throughout the germline. Despite extensive dysregulation, this mutant displays a modest reduction in total and viable brood (Albarqi and Ryder, 2021). Given the importance of MEX-3 to embryonic cell fate and the clear role of its 3′UTR to patterning its expression, we were surprised that loss of the 3′UTR did not yield a more striking reproductive phenotype. In this study, we tested the effect of stress on animals that lack the *mex-3* 3′UTR. Our results reveal that the 3′UTR is essential during thermal stress, and that animals harboring this mutation display a highly penetrant embryonic lethality phenotype that is distinct from the null allele. Our results suggest a major function of the *mex-3* 3′UTR is to buffer MEX-3 protein expression during periods of thermal stress.

## RESULTS

### The *mex-3* 3′UTR is essential at elevated temperature

**Fig. 2.**
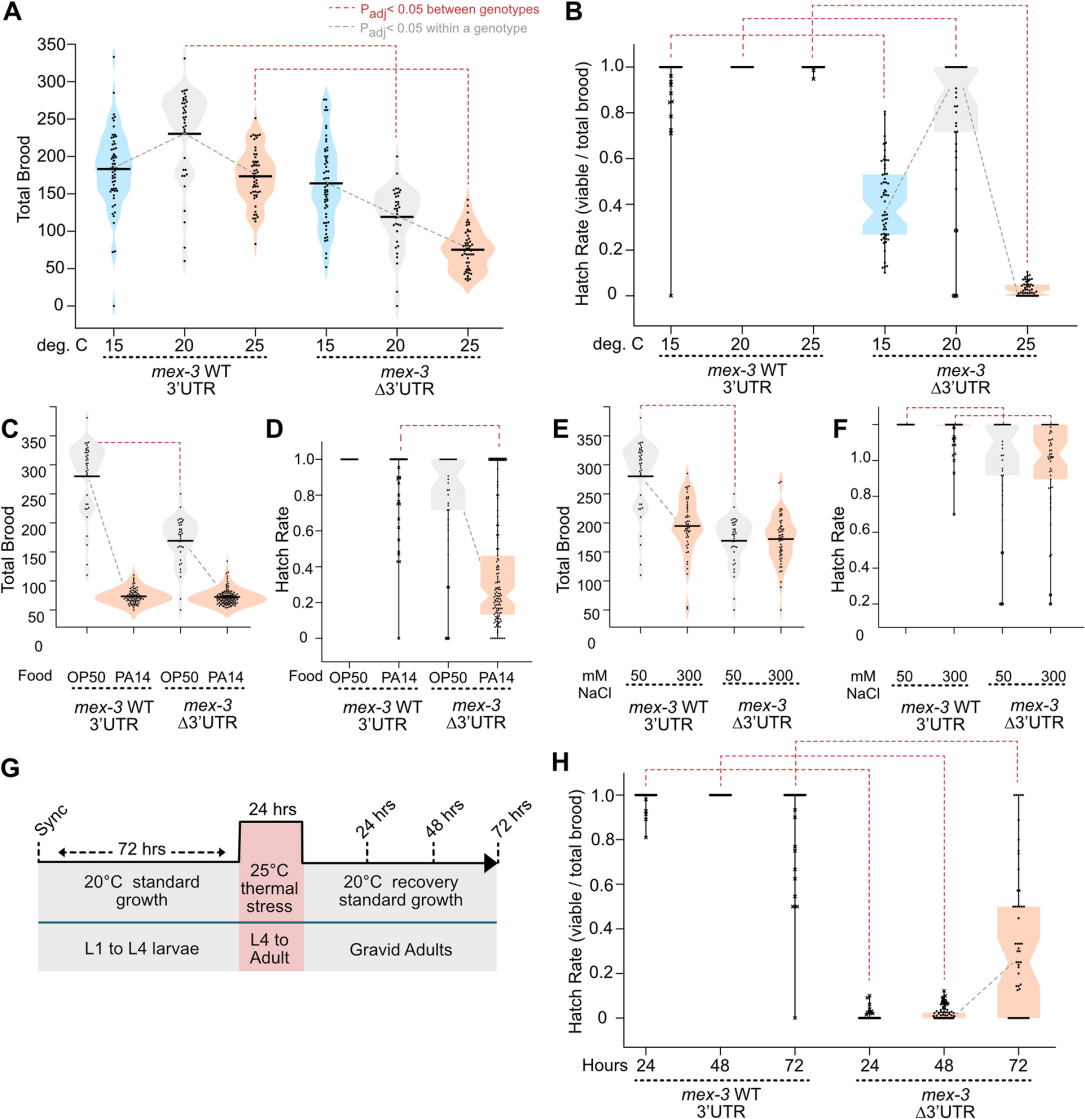
**The *mex-3* 3′UTR is essential during temperature stress.** (A) Violin plots of the total brood for control and *mex-3* 3′UTR deletion strains. Blue, gray and orange violins correspond to 15, 20 and 25°C growth, respectively. (B) Box and whisker plots depicting the hatch rate of embryos from A. Whiskers indicate the full range of the data; the box represents the range of the inner quartiles; the notch represents the median; each dot represents the hatch rate of the total brood produced from an adult hermaphrodite. Asterisks indicate outliers, as defined by the Tukey method. (C-F) Brood size and hatch rate data for infectious stress (C,D) and osmotic stress (E,F). In C,D, orange indicates *Pseudomonas aeruginosa* (PA14) while gray indicates *E. coli* (OP50) food. In E,F, orange indicates high salt (300 mM NaCl) while gray indicates standard salt (50 mM NaCl). (G) Time course to assess reversibility of the temperature-sensitive embryonic lethality phenotype. (H) Box and whiskers plot of the hatch rate post-recovery in each genotype. In all panels, gray lines indicate statistical significance within a genotype and red brackets indicate the same between genotypes in a one-way ANOVA with Bonferroni correction (*P*_adj_<0.05). All sample sizes and statistical test outcomes are listed in Table S3.

To determine whether the *mex-3* 3′UTR is important to reproductive fecundity during stress, we recorded the brood size (total number of embryos produced) and hatch rate (viable progeny/total progeny) of 3′UTR deletion mutants compared with a background-matched control under a variety of environmental stress conditions. Animals were cultured at reduced and elevated temperature (15°C and 25°C), increased osmotic stress (350 mM NaCl), and pathogenic stress induced by feeding on *Pseudomonas aeruginosa* (PA14). We compared the results to animals grown under standard laboratory conditions [20°C, 50 mM NaCl, *Escherichia coli* (OP50); Fig. 2].

The temperature dependence of the brood size produced by *C. elegans* Bristol N2 worms is bell shaped with a maximum near 18.2°C (Begasse et al., 2015). We observed a similar pattern with the wild-type (WT) *gfp::3xflag*::*mex-3*::WT 3′UTR control strain (DG4269; mean brood_WT_=183±53 at 15°C, 230±63 at 20°C and 173±36 at 25°C, *P*_adj_<0.001; Fig. 2A). By contrast, the 3′UTR deletion strain showed a linear decrease in the number of embryos produced as temperature increased, with the maximum produced at 15°C (WRM52; mean brood_Δ3′UTR_=164±55 at 15°C, 119±42 at 20°C and 75±27 at 25°C, *P*_adj_<0.001; Fig. 2A). At 15°C, both strains produced similar size broods (*P*_adj_=0.36), while at 25°C the 3′UTR deletion mutant produced less than half of the control (brood_Δ3′UTR-25_/brood_WT-25_=0.43, *P*_adj_<0.001). As such, increasing the temperature enhances the reduced fecundity phenotype nearly twofold, while lowering the temperature rescues the phenotype.

Changing the temperature also had a strong impact on the number of embryos that hatched in the *mex-3* 3′UTR deletion mutant (Fig. 2B). Consistent with our previous report, the 3′UTR deletion induced a modest reduction in the number of embryos that hatched compared to a background-matched control under standard laboratory conditions (hatch rate_Δ3′UTR_=81%, *P*_adj_=0.00158) (Albarqi and Ryder, 2021). At 15°C, only 40% of the embryos hatched (*P*_adj_<0.001), and at 25°C just 3% of the embryos hatched (*P*_adj_<0.001). By contrast, nearly all embryos produced by the control strain hatched at all three temperatures (hatch rate_WT_=96% at 15°C and 100% at 20°C and 25°C). The data show that the 3′UTR mutation induces a strong and highly penetrant embryonic lethality phenotype at 25°C, and a less-penetrant embryonic lethality phenotype at 15°C. These phenotypes are distinct from the effect on total brood size described above.

Culturing the worms on the bacterial pathogen *P. aeruginosa* (strain PA14) decreased the brood size of both strains by 90% (mean brood_WT_=26±12 on PA14, mean brood_Δ3′UTR_=22±13, *P*_adj_=1.0; Fig. 2C). However, 93% of the WT UTR embryos hatched, while only 35% of the Δ3′UTR mutant embryos hatched (*P*_adj_<0.001; Fig. 2D). As such, the 3′UTR mutation also causes a partially penetrant embryonic lethality phenotype under pathogenic stress.

Exposure to elevated sodium chloride reduced the total brood produced by both strains to a lesser extent than pathogenic stress, but there was no significant difference between genotypes (fold effect WT_300 mM/50 mM_=0.63, *P*_adj_≤0.001; fold effect Δ3′UTR_300 mM/50 mM_=0.75, *P*_adj_≤0.001; fold effect 300 mM NaCl_Δ3′UTR/WT_=0.85, *P*_adj_=0.47; Fig. 2E). Unlike thermal and pathogenic stress, exposure to high salt did not enhance the moderate embryonic lethality phenotype induced by the 3′UTR deletion mutant (fold effect Δ3′UTR_300 mM/50 mM_=0.98, *P*_adj_≤0.001; Fig. 2F). Together, the data show that some, but not all, stress conditions enhance the phenotype of the *mex-3* Δ3′UTR mutant.

The temperature-sensitive embryonic lethality phenotype is recessive. All embryos produced from balanced heterozygous worms [WRM89: mex-3 (spr9[*tn1753])/tmc20] hatched normally in the presence or absence of the 3′UTR deletion mutant (Fig. S1A,B). By contrast, mating N2 males with adult homozygous 3′UTR deletion mutant hermaphrodites did not rescue embryonic viability in the progeny (Fig. S1C,D), suggesting that changes to the maternal MEX-3 pattern are responsible for the phenotype, as has been observed with *mex-3* null mutants (Draper et al., 1996). The phenotype does not depend solely on the presence of the GFP tag, as a similar large deletion mutation [*mex-3(spr37)*] in the untagged N2 background displayed a temperature-sensitive embryonic lethality phenotype (hatch rate_20_=0.98±0.09, hatch rate_25_=0.25±0.15, *P*<0.001; Fig. S1E,F), although we note the penetrance in the GFP-tagged mutant was greater. The temperature-sensitive embryonic lethality phenotype was only observed when most of the 3′UTR was deleted. Three shorter 3′UTR deletion alleles – Δ26-167, Δ328-517 and Δ515-648 – had similar brood sizes and hatch rates to the WT 3′UTR control (Fig. S1G,H).

### The temperature-induced phenotype is reversible

MEX-3 is expressed in germline progenitor cells, in oocytes, and early-stage embryos. It plays a role in maintaining progenitor cell totipotency in the germline and it is required for anterior cell fate specification in early embryos (Ariz et al., 2009; Ciosk et al., 2004; Draper et al., 1996). Heat stress could affect one or both processes. We reasoned that if heat stress modifies MEX-3 function in embryos, the effect might be rapidly reversible upon removal from stress because new embryos are produced approximately every 20 min. By contrast, if heat stress impacts the function of MEX-3 in the germline, recovery might take more time, or affected animals may never recover.

To test these hypotheses, we isolated individual synchronized L1 larval stage worms and allowed them to mature for 72 h under standard conditions (20°C; Fig. 2G). Then, we stressed the worms at 25°C for 24 h during the late L4 to young adult stage when oogenesis begins. Worms were allowed to recover at 20°C for 24, 48 and 72 h before measuring the hatch rate of the brood deposited on each plate. Treated animals were transferred to a new plate each day so we could monitor recovery as a function of time post-stress. Our data show that the *mex-3* 3′UTR deletion mutant produced embryos with a hatch rate of 0.6% at 24 h, 1.7% at 48 h and 33% at 72 h post-thermal stress (Fig. 2H), showing partial recovery of fecundity in the window between 48 and 72 h. The WT 3′UTR control strain had a hatch rate of 99%, 100% and 94%, respectively, across the same treatment regime and time span (*P*_adj_<0.001). Our data show that, although recovery of embryonic viability is possible, it takes between 2 and 3 days post-thermal stress to restore viability, and recovery is not complete. Therefore, thermal stress likely affects some developmental process that requires days to recover.

### MEX-3 abundance is reduced in late-stage oocytes and early embryos during thermal stress

**Fig. 3.**
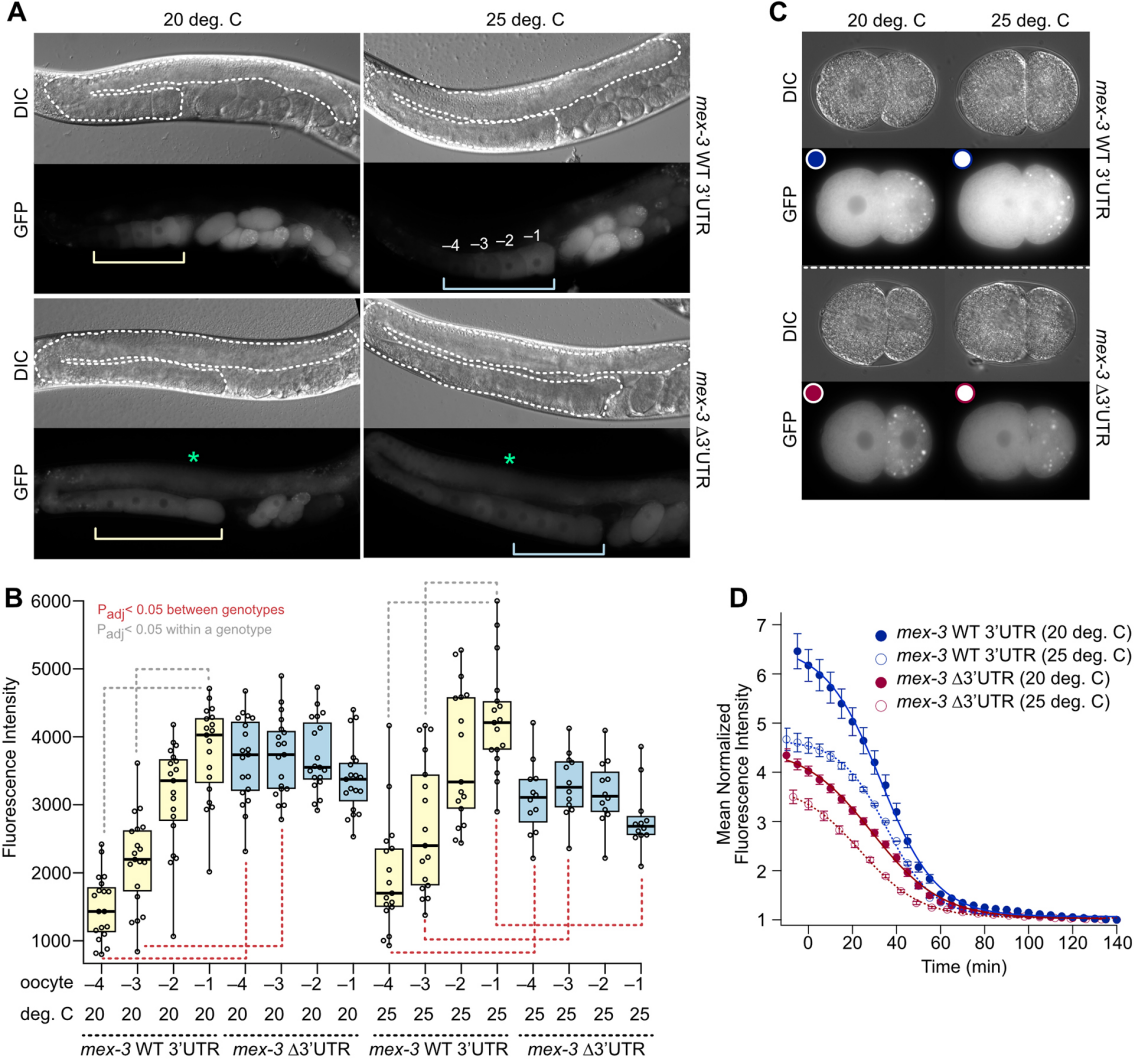
**Elevated temperature decreases GFP::MEX-3 in maturing oocytes and embryos.** (A) DIC and GFP images of the control and *mex-3* 3′UTR deletion mutant strains. The dashed white line outlines a gonad, green asterisks mark the syncytial region. The yellow and blue brackets mark developing oocytes. The −4 to −1 oocytes are labeled. (B) Box and whisker plots of oocyte fluorescence at standard and elevated temperature. Statistical significance is indicated as in Fig. 2. (C) Still frames from embryogenesis movies at t=0. The colored circles correspond to the markers in D. (D) Quantitation of GFP::MEX-3 in embryogenesis movies. The markers indicate the mean per time point of multiple movies per sample (*n*=2-5). The error bars are s.e.m., and the curves are fit to a sigmoidal equation to determine the maximal fluorescence, the half-time, and the rate of loss of the steady state GFP abundance, which incorporates both synthesis and decay. All sample sizes and statistical test outcomes are listed in Table S3.

As shown in Fig. 1, GFP::MEX-3 is expressed at low levels in the distal end of the germline and then disappears upon entry into meiosis. Expression resumes during oogenesis, peaking in the most proximal oocyte undergoing maturation. Expression increases in early embryos before the protein disappears by the 16- to 32-cell stage. We previously showed that deletion of the *mex-3* 3′UTR causes a global increase in GFP::MEX-3 protein throughout the germline, revealing that the 3′UTR acts to repress MEX-3 expression (Albarqi and Ryder, 2021). To determine whether the expression pattern is affected by temperature, we cultured control and 3′UTR deletion mutant animals at 25°C and collected images of young adult worms. The germline expression pattern of control animals did not appear to be affected by elevated temperature. Expression remained elevated throughout the germline at both temperatures in the 3′UTR deletion mutant (Fig. 3A).

Next, we quantitated GFP::MEX-3 abundance in oocytes by measuring mean pixel intensity in the four most proximal oocytes (−4 to −1; Fig. 3A). Control animals displayed the expected increase in GFP::MEX-3 abundance in older oocytes at both temperatures (Fig. 3A,B). In contrast, the expression of GFP::MEX-3 remained constant in the 3′UTR deletion mutant during oocyte development. At 25°C, the abundance of GFP::MEX-3 in the most proximal oocyte of the mutant was 30% less than controls. These results suggest that the 3′UTR contributes to increasing MEX-3 expression observed during oocyte maturation. They also suggest that MEX-3 insufficiency in oocytes contributes to the temperature-sensitive phenotype.

To measure GFP::MEX-3 expression in embryos, we filmed embryogenesis and monitored the total GFP::MEX-3 fluorescence as a function of time (Movies 1 and 2). To facilitate comparisons between embryos, we set time zero as the frame at which we observed the first cellular division post-fertilization. Total fluorescence across multiple embryos of the same genotype was averaged and fit to a sigmoidal function to determine the maximal fluorescence, the half-time, and the apparent rate of change of the protein abundance, which incorporates both synthesis and decay (Fig. 3C,D). Elevated temperature decreased the total fluorescence in embryos of both genotypes (Fmax_WT_3′UTR-20_=6.56±0.08, Fmax_Δ3′UTR-20_=4.52±0.06, Fmax_WT_3′UTR-25_=4.66±0.04, Fmax_Δ3′UTR-25_=3.75±0.05). The 3′UTR deletion strain had less maximal fluorescence at time zero than the control strain at both temperatures. Most of the visible GFP::MEX-3 was gone by 60 min in both genotypes under all conditions. However, the half-time of maximal GFP::MEX-3 abundance appeared to be shorter for 3′UTR mutant animals at elevated temperature compared with control animals (T_1/2_ WT_3′UTR-25_=35.6±0.4 min, T_1/2_Δ3′UTR-25_=23.7±0.7 min). At standard temperature, the half-times were similar between genotypes (T_1/2_WT_3′UTR-20_=32.9±0.6 min, T_1/2_ Δ3′UTR-20_=29.0±0.9 min). These results demonstrate that loss of the 3′UTR reduces the amount of GFP::MEX-3 in embryos and decreases the half-time. They also show that elevated temperature reduces GFP::MEX-3 abundance.

### Morphogenesis fails in the 3′UTR deletion mutant during thermal stress

**Fig. 4.**
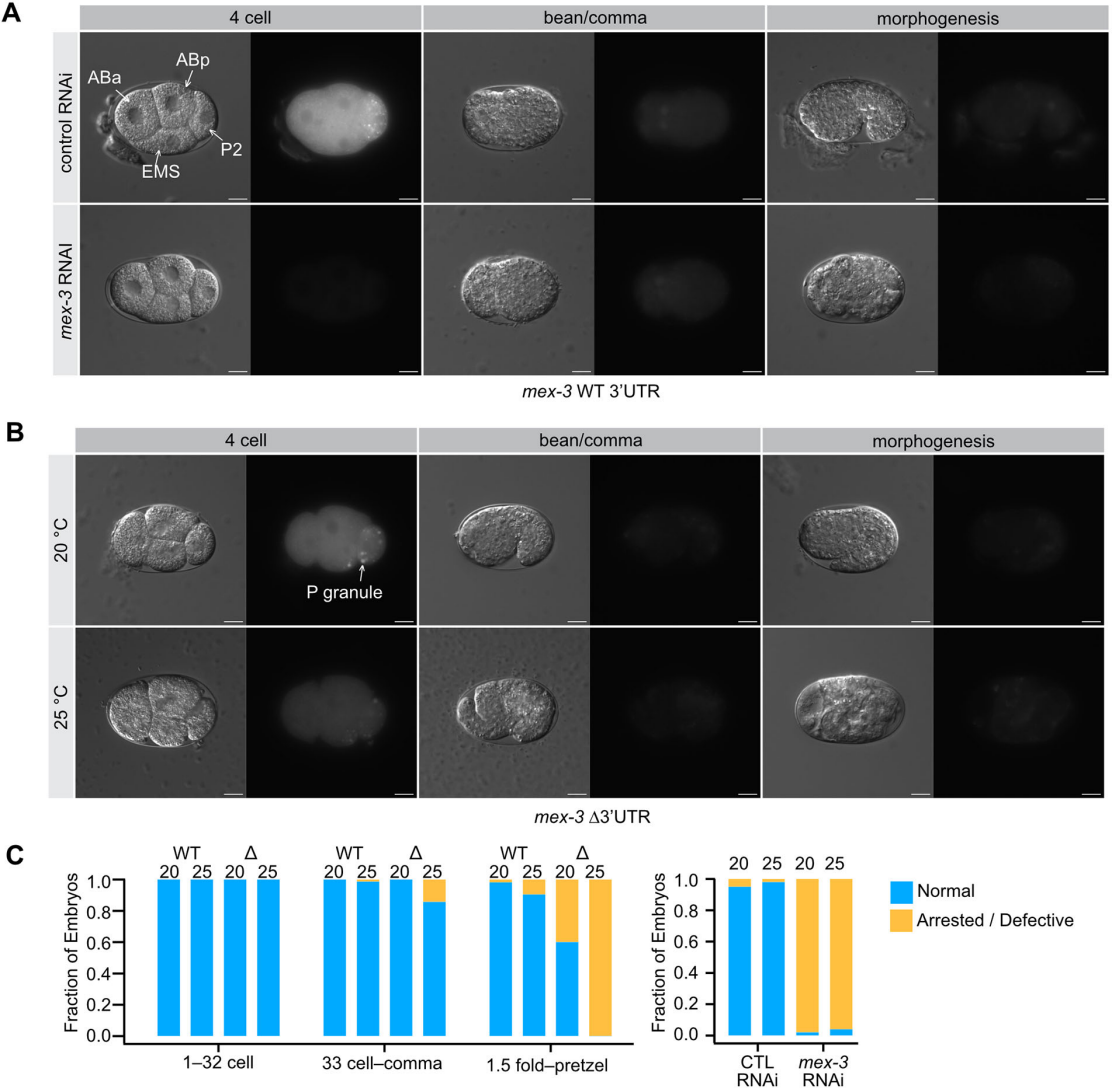
**Terminal phenotypes of *mex-3* Δ3′UTR mutants at elevated temperature.** (A) *mex-3* RNAi results in dead embryos that fail at the morphogenesis stage. The phenotype includes expanded muscle lineage cells as well as a few extra germline progenitor cells and disorganized pharyngeal tissue. (B) At elevated temperature, the *mex-3* Δ3′UTR mutants appear defective by the comma stage, and terminal embryos appear different from *mex-3* RNAi embryos. (C) Fraction of embryos that appear normal versus abnormal binned by developmental stage. For *mex-3* RNAi animals, arrest happens prior to the comma stage, so all embryos were analyzed together. All sample sizes and statistical test outcomes are listed in Table S3. Scale bars: 10 μm.

To understand the nature of the terminal phenotype, we imaged *gfp::3xflag*::*mex-3*::WT 3′UTR and *gfp::3xflag*::*mex-3*::Δ3′UTR mutant embryos as a function of embryonic stage. We compared the results to WT 3′UTR worms cultured on control RNAi or MEX-3-targeting RNAi food. GFP::MEX-3 was observed in young embryos produced from both strains grown on control RNAi food but not *mex-3* RNAi food, demonstrating effective knockdown of *mex-3* (Fig. 4A). Most embryos produced by worms cultured on control RNAi food appeared normal irrespective of genotype (WT 3′UTR=96%, Δ3′UTR=91%). As expected, most embryos produced by worms cultured on *mex-3* RNAi food arrested prior to morphogenesis, consistent with the reported null phenotype (WT 3′UTR=95% arrested, Δ3′UTR=93% arrested). Similarly, when *gfp::3xflag*::*mex-3*::Δ3′UTR embryos were grown at the permissive temperature of 20°C, embryos appeared normal until after the comma stage when morphogenesis begins (Fig. 4B,C). After that, approximately 40% of embryos appeared to arrest, forming terminal embryos with disorganized tissue and zones of apoptosis. The phenotype became more penetrant at elevated temperature, with 100% of late-stage embryos appearing abnormal while the younger embryos appeared normal (Fig. 4B,C). Interestingly, we noted visual differences between the terminal embryos produced by *mex-3* RNAi compared to the *mex-3* Δ3′UTR deletion mutant, suggesting that the terminal phenotype may be different.

**Fig. 5.**
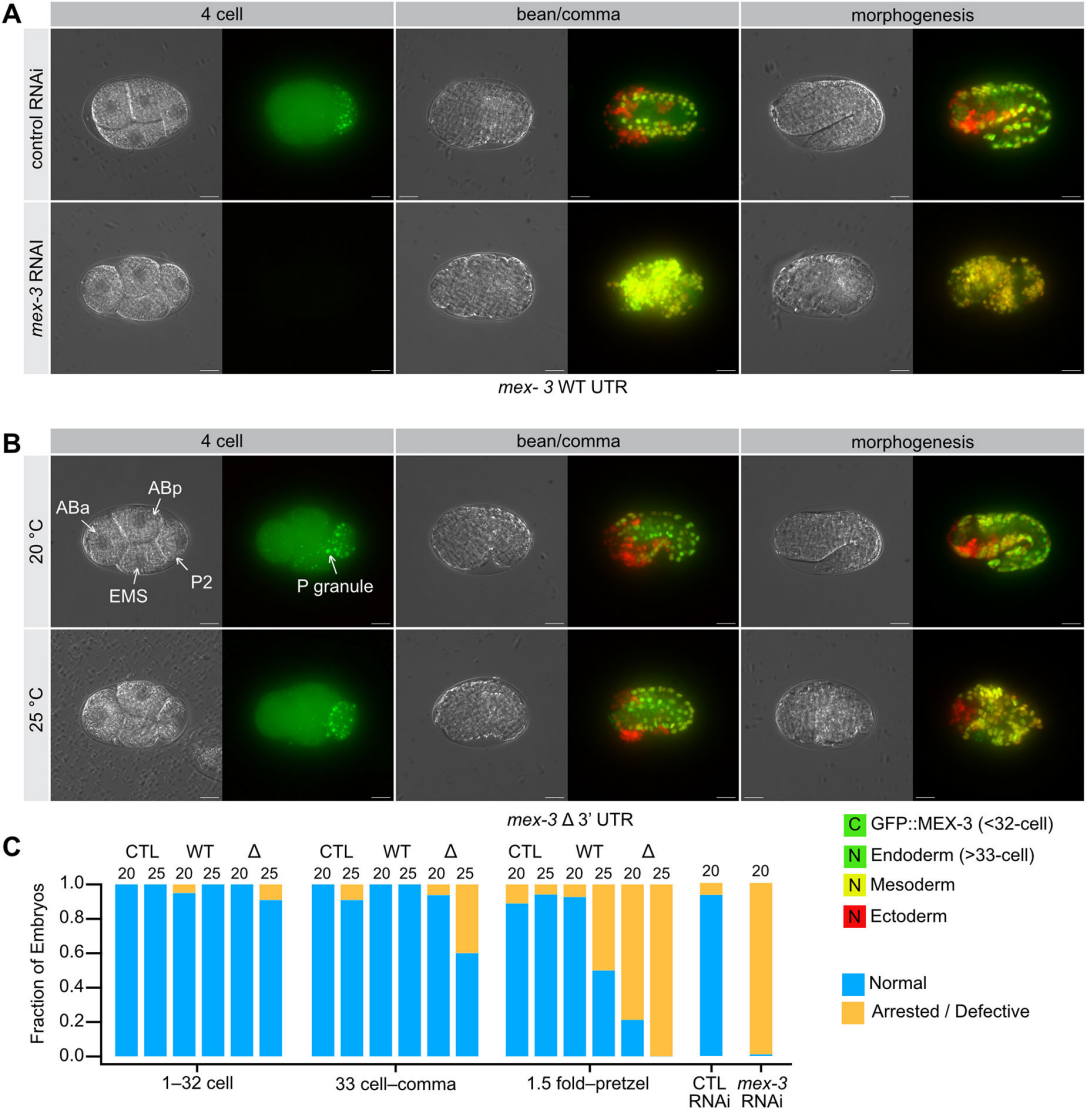
**Germ layer patterning defects in the *mex-3* Δ3′UTR mutant.** (A) *mex-3* RNAi results in dead embryos with a large expansion of muscle lineage cells (mesoderm, yellow). Control animals develop normally, with all three germ layers present and appropriately patterned. Cytoplasmic (C) green represents GFP::MEX-3 protein, nuclear (N) green indicates endoderm, nuclear (N) yellow indicates mesoderm and nuclear (N) red indicates ectoderm. (B) At elevated temperature, the *mex-3* Δ3′UTR mutants form all three germ layers, but patterning appears defective with a moderate expansion of muscle lineage cells (yellow). (C) Fraction of embryos that appear normal versus abnormal binned as in Fig. 4. The genotype and the temperature of growth are shown. CTL is a control for the germ layer background (strain OD1854). All sample sizes and statistical test outcomes are listed in Table S3. Scale bars: 10 μm.

To better compare the extent of differentiation in the terminal phenotypes, we crossed the GFP::*mex-3*::Δ3′UTR mutant and the WT control into OD1854, a germ layer reporter strain. This strain expresses a *Ppha-4*::*pha-4::gfp::pha-4* 3′UTR marker in the endoderm (pharynx and intestine; Fig. 5, green), *Phlh-1::gfp::his-72* and *Phlh-1:mCherry::his-72* reporters in mesoderm (body wall muscle; Fig. 5, yellow) and *Pdlg1-7I*::*mCherry::his-72* and a *Pcnd-1*::*mCherry::his-72* in the ectoderm (epidermis and about one third of neurons; Fig. 5, red) (Wang et al., 2019). In addition to providing a clear view of all three germ layers, the mesoderm reporter specifically marks body wall muscle cells, which have been shown to be expanded in *mex-3* null mutants (‘mex’ stands for muscle excess) (Draper et al., 1996).

Under standard growth conditions, embryos with the GFP::*mex-3*::WT 3′UTR allele in the germ layer reporter background developed normally. GFP::MEX-3 can be readily distinguished from the transgenic germ layer reporters because (1) its expression is cytoplasmic whereas the reporter transgenes are nuclear, and (2) GFP::MEX-3 disappears prior to transgene expression after zygotic gene activation. Culturing this strain on *mex-3* RNAi food caused a strong and highly penetrant expansion of mesoderm in the terminal embryos (Fig. 5A; 100% of embryos). By contrast, 96% of the embryos produced by worms cultured on control RNAi appeared normal with all three germ layers present. Embryos produced by this strain appeared normal under standard growth conditions and at 25°C.

In contrast, worms with the *gfp::3xflag*::*mex-3*::WT 3′UTR allele displayed germ layer patterning defects in half of the embryos by the 1.5-fold stage or older at elevated temperature, suggesting the germ layer reporter background causes a synthetic phenotype with GFP::MEX-3. However, the Δ3′UTR mutant showed a much more penetrant germ layer patterning phenotype that manifested earlier and at both temperatures (Fig. 5B,C). At 20°C, patterning of the Δ3′UTR mutant appeared normal until the 1.5-fold stage wherein 80% of the embryos appeared defective. At 25°C, 100% of older Δ3′UTR mutant embryos displayed the patterning phenotype, and 40% of younger embryos between the 32-cell stage and the comma stage also appeared defective. Interestingly, the germ layer patterning phenotype of the Δ3′UTR mutant was different from the *mex-3* RNAi phenotype. All three germ layers were visible, but the pattern was disorganized, and the mesoderm expansion was not as pronounced. As such, the UTR deletion allele potentially retains some MEX-3 activity in the embryo, despite reduced abundance, and progresses further before arresting compared to *mex-3* RNAi-treated animals.

### The number of germline progenitor cells increases in the 3′UTR deletion mutant

**Fig. 6.**
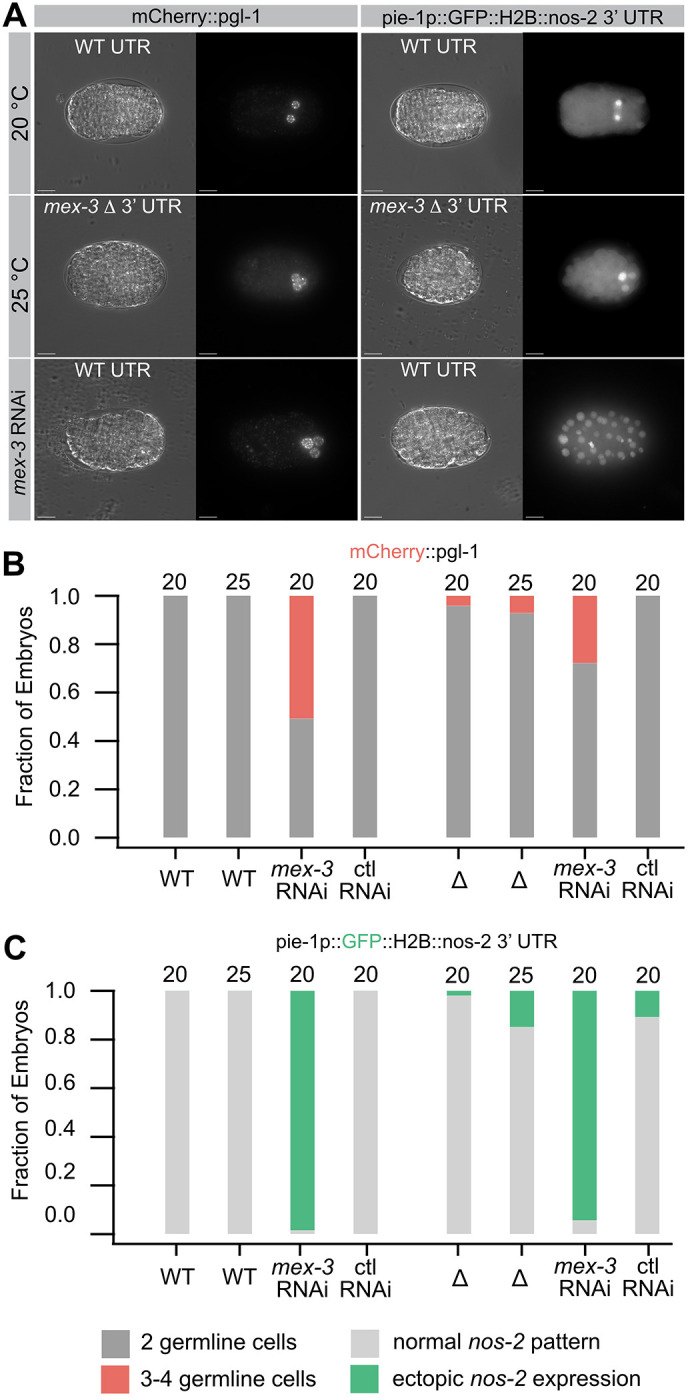
**Germline progenitor cell expansion in the *mex-3* 3′UTR deletion.** (A) Two different germline progenitor cell markers, mCherry::*pgl-1* and pie1p::GFP::H2B::*nos-2* 3′UTR, were used to evaluate the number of germline progenitor cells in *mex-3* WT and 3′UTR deletion embryos. MEX-3 directly regulates *nos-2* through its 3′UTR, so expansion to all cells in *mex-3* RNAi is consistent with de-repression (D'Agostino et al., 2006). (B) Quantification of the fraction of embryos displaying three or four germline progenitor cells in the mCherry::*pgl-1* background. (C) Fraction of embryos with more than two germline progenitor cells in pie-1p::GFP::H2B::*nos-2* 3′UTR. reporter. All sample sizes and statistical test outcomes are listed in Table S3. Scale bars: 10 µm.

A second key feature of the *mex-3* null mutant phenotype is an increase in the number of germline progenitors. In normal animals, two germline progenitors (Z2 and Z3) are produced by the 88-cell stage (Wang and Seydoux, 2013). In *mex-3* null mutants, three or four germline progenitor cells are observed (Draper et al., 1996). To assess whether the Δ3′UTR mutant causes a similar phenotype, we tagged the germline-specific *pgl-1* gene with mCherry at the N terminus in both the WT and UTR deletion mutant strains (Kawasaki et al., 1998) (Fig. 6A). We note that the *mex-3* WT 3′UTR strain had the expected number of two germline progenitors at 20 or 25°C. By contrast, the 3′UTR deletion mutation showed an expanded progenitor cell phenotype in some embryos, but with low penetrance (4% of embryos at 20°C, 7% of embryos at 25°C; Fig. 6B). When the WT 3′UTR strain was cultured on *mex-3* RNAi food, 51% of embryos showed an expansion of germline progenitors, while none of the embryos showed expanded progenitors on control RNAi food. The results show that the 3′UTR mutant can produce the same phenotype as the null, but with low penetrance at both temperatures. This result is consistent with the model that the *mex-3* 3′UTR deletion mutant retains some MEX-3 activity in embryos despite reduced expression.

### The MEX-3 target mRNA *nos-2* remains repressed during thermal stress

MEX-3 translationally represses the expression of the germline cell fate determinant *nos-*2 (D'Agostino et al., 2006). We previously showed that MEX-3 regulates a *nos-2* 3′UTR reporter transgene in somatic cells by binding to two MEX-3 recognition elements (MREs) in the *nos-2* 3′UTR (Pagano et al., 2009). In that study, *mex-3* RNAi led to ectopic expression of the reporter transgene in all cells of the embryo.

To test whether the *nos-2* reporter remains repressed when the *mex-3* 3′UTR is deleted, we crossed the *nos-2* reporter transgene into the *mex-3* WT 3′UTR strain and the *mex-3* Δ3′UTR strain. We observed ectopic expression limited to three or four cells in 2% of embryos produced by the 3′UTR deletion mutant embryos cultured at 20°C (Fig. 6A,C). This number increased to 15% at 25°C. By contrast, we observed no ectopic expression in the WT UTR strain at either temperature. We note that expression was not observed in all cells of the early embryo but was instead restricted to three or four germline progenitor cells. This contrasts with the *mex-3* RNAi phenotype. Consistent with our previous report, when we cultured either strain on *mex-3* RNAi food, we observed ectopic expression in all embryonic cells in 95% of *mex-3* WT 3′UTR embryos and 98% of *mex-3* Δ3′UTR embryos (Fig. 6A,C) (Pagano et al., 2009). Embryos produced by animals cultured on control RNAi food developed normally. The results suggest that *nos-2* remains repressed in the *mex-3* Δ3′UTR strain in the majority of somatic blastomeres at both temperatures. Although we did see an increase in the number of embryos with limited ectopic expression of the *nos-2* reporter, this could be explained by the increase in the number of germline progenitor cells produced in this mutant, as noted with the *mCherry::pgl-1* marker (Fig. 6A). The results reveal another difference between the *mex-3* 3′UTR deletion mutant and the *mex-3* null phenotype, suggesting that the former retains some MEX-3 activity in the embryo despite decreased abundance and the embryonic lethality observed at 25°C.

### Dysregulation of *mex-3* impacts multiple gene categories

**Fig. 7.**
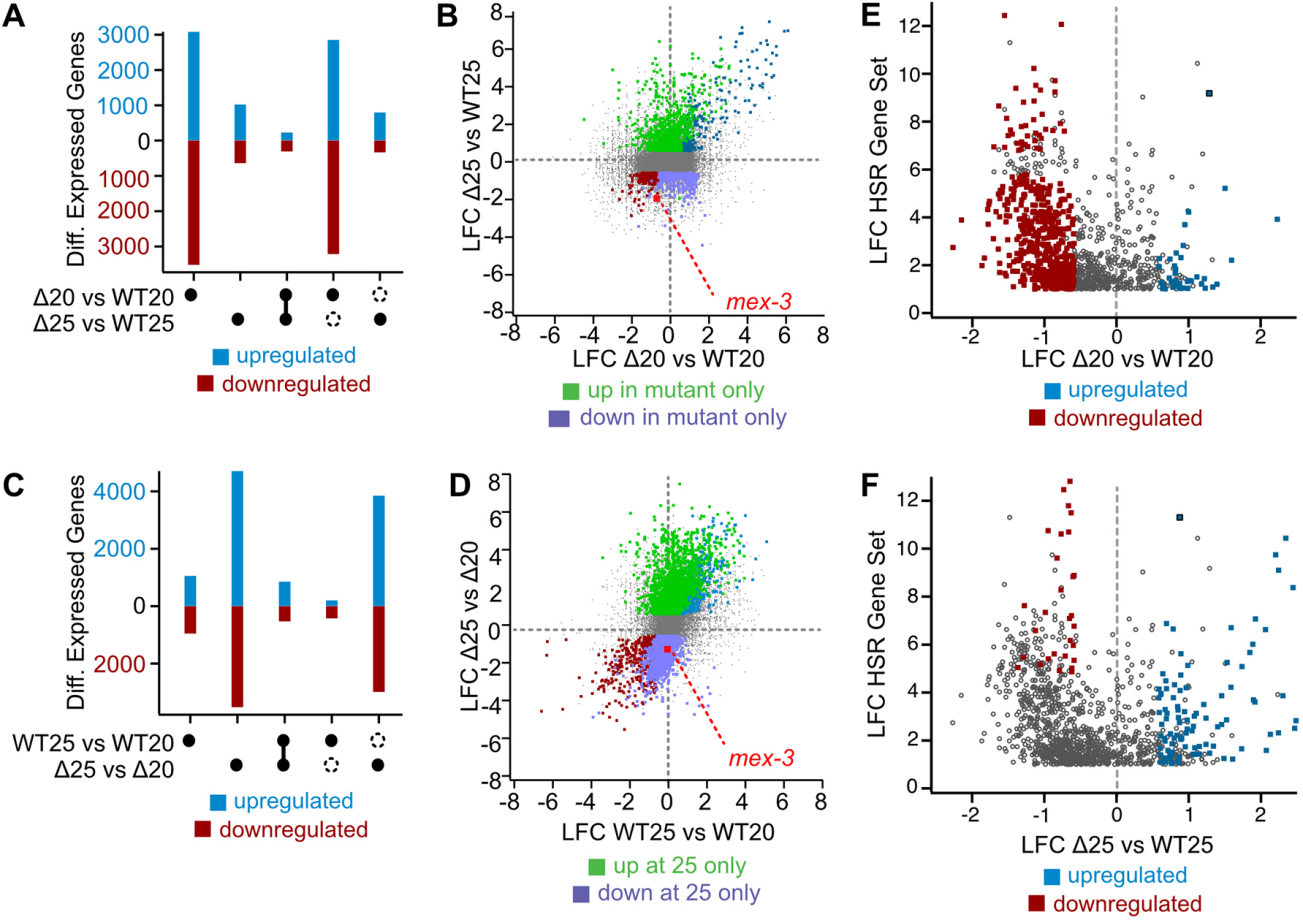
**RNA-seq analysis as a function of temperature.** (A) UpSet plot comparing genotype-responsive changes at both temperatures. The black circles indicate the data sets analyzed. Connected circles are the intersection of the two groups. Open circles represent data present in one group, but absent in the other. Upregulated genes are in blue, downregulated genes are in red. (B) Comparison of the log_2_ fold changes (LFC) in expression as a function of genotype. The colored circles are defined by the legend. (C) UpSet plot comparing temperature-driven changes in each genotype. Labels are as in A. (D) Comparison of the log_2_ fold changes in expression as a function of temperature. Labels are as in B. The red dot indicates *mex-3* transcripts. (E) Comparison of the genotype dependent changes in the *mex-3* Δ3′UTR mutant to a high confidence list of heat shock-responsive genes. The colors are as defined in the legend. Gray circles represent heat shock-responsive genes that are unchanged in the 3′UTR mutant, or are not statistically significant in our data set (*P*_adj_<0.05). All sample sizes and statistical test outcomes are listed in Table S4.

To gain insight into how the 3′UTR deletion mutant impacts gene expression, we used RNA sequencing (RNA-seq) to measure the transcriptome of mutant and control strains at both permissive (20°C) and restrictive (25°C) temperatures (Fig. 7A,B). We used DESeq2 to identify differentially expressed genes between the four groups, comparing data for three biological replicates for each strain at each condition (Love et al., 2014).

First, we compared the *mex-3* 3′UTR deletion mutant to the control strain cultured at 20°C. This analysis identified 3082 upregulated and 3520 downregulated transcripts with a log_2_ fold-change threshold of 0.585 and a *P*_adj_ value less than or equal to 0.05 (Fig. 7A). When the temperature was increased to 25°C, the number of affected genes dropped to 1025 upregulated and 638 downregulated transcripts, presumably due to the increase in gene expression noise at higher temperature. Most of the gene expression changes observed in the mutant at 25°C were not observed at 20°C, with 78% (795/1025) of upregulated genes and 52% (333/638) of downregulated genes unique to the mutant strain at elevated temperatures (Fig. 7A,B). This is not surprising considering that the embryonic lethality phenotype is only prevalent at elevated temperature. We note that one of the downregulated transcripts is *mex-3* itself, suggesting that the reduced GFP::MEX-3 protein expression observed in oocytes and embryos at the elevated temperature (Fig. 3) correlates with reduced *mex-3* transcript abundance.

### The *mex-3* 3′UTR mutant limits the heat-shock response

Next, to identify temperature responsive differences in the *mex-3* 3′UTR deletion mutant, we reanalyzed the data comparing genotype matched samples grown at 25°C directly to samples grown at 20°C (Fig. 7C). The data reveal 4707 upregulated and 3518 downregulated transcripts with a log_2_ fold-change threshold of 0.585 and a *P*_adj_ value less than or equal to 0.05. We also identified 1057 upregulated and 954 downregulated transcripts in the control strain when comparing 25°C to 20°C samples directly using the same filtering criteria. Somewhat surprisingly, there is little overlap between the temperature-responsive transcriptome in each strain (Fig. 7D). Only 18% (856/4707) of upregulated transcripts and 15% (530/3518) of downregulated transcripts were observed in both genotypes. As such, the *mex-3* 3′UTR deletion mutant appears to broadly change which transcripts are responsive to elevated temperature, suggesting that the temperature-responsive gene expression program is somehow dysregulated in this mutant.

To better understand which gene sets are dysregulated as a function of temperature, we performed a WormCat gene ontology analysis (Fig. S2) (Holdorf et al., 2020). The analysis revealed low correspondence between the temperature-responsive gene sets between strains. In the control strain, we observed downregulation of metabolism and proteolysis gene sets, consistent with a mild stress response. We also observed downregulation of the extracellular materials gene set and the stress response gene set. Upregulated gene categories were limited to pseudogenes and unassigned genes.

In contrast, the *mex-3* 3′UTR deletion mutant showed changes in numerous gene categories at elevated temperature. The largest and most significant downregulated gene sets in the mutant were related to cell cycle, mRNA functions, and ribosomes. The upregulated sets included genes related to neuronal function, signaling, and transmembrane transport. The number and variety of altered gene sets suggests that MEX-3 dysregulation impacts numerous gene pathways. Consistent with this idea, MEX-3 has previously been shown to be important for maintenance of totipotency in the germline, limiting expression of somatic cell fates in the germline, including inhibition of neuronal lineages (Ariz et al., 2009; Ciosk et al., 2006).

Because of the wide variety of changes, we wondered whether the global response to temperature stress was altered in the *mex-3* mutant. We compared the changes in gene expression observed in the *mex-3* 3′UTR deletion mutant to the canonical set of genes upregulated in response to heat shock. A high-confidence set of 1060 protein-coding genes that are upregulated in WT (N2) worms when treated with a heat shock (35°C for 4 hours) has been published (Schreiner et al., 2019). We observed that 546 (52%) of these genes were downregulated in the *mex-3* 3′UTR deletion mutant compared to the genotype matched control worms when grown at standard temperature (Fig. 7E). By contrast, only 42 (4%) were upregulated. As such, approximately half of the heat shock-responsive genes were expressed at a lower basal level in the *mex-3* 3′UTR deletion mutant than in control worms. At 25°, we saw fewer downregulated (3%; Fig. 7F) and more upregulated (10%) heat-shock genes in the mutant compared to control. This is consistent with the fewer overall changes observed at high versus low temperature growth, and the data at this temperature are complicated to interpret due to the extensive embryonic lethality. The data suggest that MEX-3 helps to establish the baseline heat-shock response, and that reduced expression of these genes may contribute to the embryonic lethality observed upon mild temperature increase.

## DISCUSSION

In this study, we show that a *mex-3* 3′UTR deletion mutant has a strong and highly penetrant embryonic lethal phenotype when cultured at a temperature of 25°C. This phenotype is characterized by reduced GFP::MEX-3 levels in maturing oocytes and embryos, failure to undergo morphogenesis, and an expanded number of embryonic germline progenitor cells. Unlike *mex-3* RNAi embryos, the 3′UTR deletion mutant embryos cultured at elevated temperature generate all three germ layers and show less pronounced expansion of body wall muscle. Moreover, a germline-specific *nos-2* 3′UTR reporter remains repressed in somatic blastomeres in the embryos. The results suggest that the 3′UTR deletion mutant is likely a temperature-sensitive hypomorphic allele, with reduced MEX-3 activity in embryos that remains sufficient to promote differentiation beyond what is observed with a *mex-3* null allele, but insufficient to support viability.

This result is surprising because the most obvious phenotype of the *mex-3* 3′UTR deletion under standard growth conditions is increased GFP::MEX-3 expression throughout the germline (Albarqi and Ryder, 2021). This suggests two things. First, reduced MEX-3 abundance is worse for reproductive fecundity than elevated expression. Second, a major role of the *mex-3* 3′UTR is to ensure robust MEX-3 expression at the oocyte-to-embryo transition in variable environmental conditions. Although animals can thermoregulate by moving to a more suitable environment, embryos cannot, so it is reasonable to hypothesize that the worm has evolved a mechanism to ensure that a key cell fate determinant is expressed under a variety of environmental conditions.

### Repressing and activating circuits in the *mex-3* 3′UTR

The results presented here suggest that the *mex-3* 3′UTR contains at least two regulatory circuits that act in different regions of the germline. The first is a repressive circuit that ensures *mex-3* silencing in the meiotic region of the germline before re-cellularization into oocytes. The second is an activating circuit that functions in maturing oocytes and early embryos that boosts MEX-3 levels at the oocyte-to-embryo transition. The repressive circuit does not appear to be temperature sensitive, as de-repression is observed in all conditions in the *mex-3* 3′UTR mutant animals. Failure of the activating circuit appears to be exacerbated by elevated temperature, as mutant animals cultured at 25°C have less GFP::MEX-3 in the most proximal oocyte and in embryos than WT controls or mutants cultured at standard temperature. While it remains formally possible that de-repression is due to transcriptional upregulation, we observe a decrease rather than an increase in the steady state abundance of *mex-3* mRNA in the RNA-seq data at both temperatures (Fig. 7B). This observation, coupled to the deletion being within the 3′UTR of the gene, suggests that de-repression is due to mechanisms acting at the post-transcriptional level.

The *trans* factors that govern *mex-3* 3′UTR-dependent repression and/or activation are not known, but a few candidates arise from inspection of known RBP motifs in the *mex-3* 3′UTR. GLD-1 is a STAR/KH domain RBP that binds with high affinity and specificity to a heptanucleotide motif in its target RNAs (Jungkamp et al., 2011; Ryder et al., 2004; Wright et al., 2011). Its expression increases at the switch from mitosis to meiosis, then decreases during oogenesis (Jones et al., 1996). There is a strong GLD-1 binding motif (GBM) in the *mex-3* 3′UTR (Ryder et al., 2004; Wright et al., 2011). This interaction has been confirmed by high-throughput GLD-1 interactome studies (Jungkamp et al., 2011; Wright et al., 2011). The anticorrelated pattern of expression, the presence of a high-affinity consensus binding motif, and the known role of GLD-1 in translationally repressing other germline transcripts make it a strong candidate for a *trans*-acting factor that represses *mex-3* in the meiotic germline. However, a 134 base pair deletion that removes the high-affinity GBM does not result in an abnormal phenotype and does not display altered expression (Albarqi and Ryder, 2021). As such, additional motifs, potentially recognized by other factors, may also be required or may be redundant with the GBM.

The *trans-*acting factors that govern activation in oocytes and embryos are harder to predict. In addition to a strong GBM, the *mex-3* 3′UTR harbors three consensus binding motifs for POS-1, three for FBF-1 and three motifs for MEX-3 itself (MREs) (Bernstein et al., 2005; Farley et al., 2008; Kalchhauser et al., 2011; Pagano et al., 2009; Wang et al., 2009). The 3′UTR deletion allele removes all three *mex-3*-binding sites, all three POS-1 sites, and one of the FBF sites. POS-1 is a tandem zinc finger RBP expressed exclusively in early embryos after fertilization (Tabara et al., 1999). As such, this protein cannot be responsible for *mex-3* activation in late-stage oocytes. Similarly, FBF is an RBP that is expressed in mitotic progenitor germline progenitor cells where it promotes progenitor fate renewal (Zhang et al., 1997). It is not found in late-stage oocytes and is unlikely to contribute to enhanced expression.

The remaining candidate is MEX-3 itself. It is intriguing to consider the possibility that MEX-3 might activate its own expression. The three MEX-3-binding sites are in distant regions of the 3′UTR, which could explain why most of the 3′UTR must be removed to observe the phenotype. MEX-3 abundance increases as GLD-1 decreases in oocytes. The GBM is adjacent to two of the MREs, suggesting the potential for competition between MEX-3 and GLD-1 for binding to adjacent binding motifs. It remains equally likely that other RBPs, binding to yet to be determined *cis*-regulatory motifs, govern increased GFP::MEX-3 abundance in oocytes. Some of the binding sites are in conserved regions of the *mex-3* 3′UTR, while others are not, and there are several conserved regions of the 3′UTR that lack known RBP motifs (Fig. S3). More work will be needed to identify the functional elements within the 3′UTR responsible for the phenotypes presented here.

### What is the basis of temperature sensitivity?

The molecular mechanism that governs the temperature sensitivity of the *mex-3* 3′UTR deletion phenotype is not known. Temperature-sensitive mutations are often found within the protein-coding sequence of genes in which the mutation alters protein-folding stability (Sandberg et al., 1995). Here, the mutation is present in the 3′UTR and does not perturb the protein-coding sequence. As such, the temperature sensitivity must arise by a different mechanism.

The RNA-seq data reveal a few plausible mechanisms. First, a large fraction of the high-confidence heat shock-response genes is downregulated in the *mex-3* 3′UTR mutant. It could be that dysregulation of *mex-3* activity leads to attenuation of the heat-shock response by a direct or indirect mechanism. Loss of the ability to adapt to heat could potentially explain the temperature-sensitive phenotype. HSF-1 is a conserved transcriptional regulator of the heat-shock response (Hajdu-Cronin et al., 2004). In *C. elegans*, monomeric HSF-1 is held in an inactive state by forming a complex with DDL-1/2 and HSB-1 (Chiang et al., 2012). Upon experiencing heat stress, HSF-1 is released from this complex and forms a homotrimer that binds to heat-shock elements found in the promoters of many heat-response genes Intriguingly, we note that *ddl-1* and *ddl-2* transcripts are both upregulated in the *mex-3* 3′UTR mutant, suggesting that perhaps MEX-3 controls the abundance of these negative regulators of the heat-shock response through some alternative mechanism, perhaps through direct regulation of their mRNA stability, or control of some upstream component that feeds into the HSF pathway. More work will be needed to determine whether these factors or others contribute to the embryonic lethality phenotype.

Second, our data show that many ribosomal protein gene transcripts are downregulated in the *mex-3* 3′UTR mutant at elevated temperature. This suggests that increasing temperature leads to a coordinated decrease in ribosome biogenesis, which could broadly impact maternal gene translation in the embryo, including MEX-3 itself, as observed in Fig. 3. Ribosome biogenesis is essential to successful reproduction, and maternally supplied ribosomes are sufficient for successful embryogenesis (Cenik et al., 2019). Ribosome protein gene biosynthesis is also necessary for mitochondrial ribosome function and mitochondrial health. A more recent study demonstrated that haploinsufficiency of five different ribosomal protein genes alters mitochondrial morphology, and haploinsufficiency of *rps-10* has broad effects on mitochondrial function and cellular energy states (Surya et al., 2024 preprint). Several studies show that phosphorylation of eEF2 by eEF2 kinase and/or AMPK impacts translation rate and thermogenesis in mammals (Knight et al., 2015; Xie et al., 2019; Yamada et al., 2019). Intriguingly, *aak-1* but not *aak-2* (AMPK homologs) or *eefk-2* (eEF2K homolog) is upregulated in our RNA-seq data. More work will be needed to determine whether these genes and/or others contribute to the temperature-sensitive phenotypes. We note that ribosomal protein genes and metabolism genes were enriched in our WormCat ontology analysis in the mutant at elevated temperature. How MEX-3 impacts ribosome biogenesis and activity is not known.

Many additional models consistent with these data could be drawn. While it is tempting to speculate that specific gene expression changes that manifest at 25°C in the mutant strain are responsible for the temperature-sensitive phenotype, it remains equally likely that these changes are a consequence, rather than a cause of the phenotype. It is intriguing to speculate that a unifying molecular mechanism connects all the stress-response phenotypes observed. However, our study does not shed light on the genes responsible for the cold sensitivity and pathogen-induced stress phenotypes, and considerably more work will be necessary to map the genetic and molecular pathways responsible for these phenotypes.

### Temperature sensitivity, developmental processes, and post-transcriptional regulation

Post-transcriptional regulatory pathways have been shown to be crucial for biological robustness in other aspects of nematode development as well (Burke et al., 2015; Ilbay and Ambros, 2019). Specifically, miRNAs that repress key heterochronic pathway mRNA targets during larval development rarely have a phenotype when mutated (Alvarez-Saavedra and Horvitz, 2010; Miska et al., 2007). This is in part due to the redundancy built into miRNA families: multiple miRNAs in the same family recognize the same seed sequence and can thus act redundantly. Also, multiple miRNAs can regulate the same mRNA through distinct, yet functionally redundant, binding sites. As such, their importance could have been easily overlooked. The Abbott lab identified strong miRNA mutant phenotypes in sensitized genetic backgrounds (Brenner et al., 2010). Expanding upon this study, the Ambros lab demonstrated that multiple miRNA family mutants display enhanced phenotypes upon oscillating temperature stress but not upon other forms of stress (Burke et al., 2015; Ilbay and Ambros, 2019).

The results presented here show that the *mex-3* 3′UTR is essential for embryonic morphogenesis at elevated temperature. It is appealing to speculate that a major role for the masked ‘load’ of maternal mRNAs first described by Spirin in 1966 (Spirin, 1966) is to act as a buffer to ensure sufficient maternal protein is produced in uncertain environments. Whether the results presented here are generalizable to other maternal transcripts remains to be seen. Few large 3′UTR deletion alleles in maternal transcripts have been engineered to date, so a comprehensive assessment cannot be made at this time.

## MATERIALS AND METHODS

### Strains and nematode culture

All strains used in this study are listed in Table S1. Strains were maintained by growing animals on nematode growth medium (NGM) seeded with *E. coli* (OP50) under standard conditions (Stiernagle, 2006). All primers used to produce or evaluate the strains are listed in Table S2. WRM75 was generated by crossing WRM52 males with OD1854 (germ layer reporter) hermaphrodites, selecting heterozygous male cross progeny, then back-crossing the F1 males to strain OD1854 before isolating individuals and following progeny, confirming the desired genotype by PCR and fluorescence imaging. WRM77 was made by the same approach using DG4269 males.

WRM79 was generated by crossing WRM52 males with WRM1 (*nos-2* 3′UTR reporter) hermaphrodites, then backcrossing the F1 males to strain WRM1 before isolating individuals and following progeny, confirming the desired genotype by PCR. WRM80 was made by the same approach using DG4269 males.

WRM81 and WRM82 (*pgl-1* reporter strains) were generated using CRISPR/Cas9 following the procedure of Ghanta and Mello (Ghanta et al., 2021; Ghanta and Mello, 2020). To produce WRM81, WRM52 worms were injected with an RNP mixture containing *Streptococcus pyogenes* Cas9 (IDT, 1081058; 10 µg/µl), tracrRNA (0.4 µg/µl), crRNA (0.4 µg/µl; gRNA sequence: 5′-GUUUCAUCCAUUUCACAUGG-3′), 500 ng of melted dsDNA recombination template (25 ng/µl final concentration), PRF::rol-6 plasmid (500 ng/µl) and nuclease-free water. The recombination template was made by PCR amplifying the recombination template from a plasmid template (pCCM953, a gift from Craig Mello, UMass Chan Medical School, Worcester, MA, USA). The PCR product was purified using a kit (Zymo Research DNA Clean & Concentrator™, D4034) The desired genotype was confirmed by PCR and sequencing. WRM82 was made by injecting DG4269 animals with the same injection mix.

WRM113 was made using a similar approach, except N2 hermaphrodites were injected with an RNP mixture containing Cas9, tracrRNA, two crRNA sequences (WRM113_guide1: 5′–GAGAGUCUACACGAUAGUAA–3′; WRM113_guide2: 5′–CCAUUUUCUACUUUGUUCAU–3′), and an ssODN oligonucleotide donor template (WRM113_ssodn; Table S2). This mutant recapitulates the *spr9* allele in that the mutant deletes nucleotides 28-651, but the sequence 5′-TTCATTCCAATT-3′ is inserted between the break points. We assume this sequence was added during repair of the lesion, as it was not included in our repair template. This allele is named *spr37*.

All strains generated in this study will be made available to other researchers through the *Caenorhabditis* Genetics Center or by request to the corresponding author.

### Brood size and hatch rate assays

Brood size and hatch rate measurements were conducted in triplicate for each condition and genotype. Strains were synchronized by bleaching adult worms in a 20% alkaline hypochlorite solution (3 ml concentrated Clorox bleach, 3.75 ml 1 M filtered sodium hydroxide, 8.25 ml filtered MilliQ H_2_O), embryos were recovered by centrifugation (2655 ***g*** for 1 min), then washed extensively in M9 buffer before they were allowed to hatch overnight. Once synchronized as starved L1 larvae, animals were transferred onto NGM agar plates seeded with OP50 and allowed to grow to the L3/L4 stage at room temperature. For each replicate, 20 individual L4 animals were separated onto individual NGM plates then introduced to a stress condition (15°C, 25°C, 300 mM NaCl, *P. aeruginosa* PA14) or a standard growth condition control (20°C, 50 mM NaCl, *E. coli* OP50). After 24 h, each animal was transferred to a fresh plate, the total number of embryos laid was counted, then both plates were re-introduced to the stress condition. After another 24 h, the number of hatched larvae was also counted from the first plate. The process was repeated until the animals no longer produced fertilized embryos (∼7 days post-synchronization). To measure recovery from 25°C stress, assays were performed as above but worms were placed in the stress condition for 24 h, then removed to 20°C for the remainder of the experiment.

From these data, the total number of embryos produced, the total viable progeny produced, and the hatch rate (fraction of viable embryos) were determined for each genotype, condition, or recovery period. Statistical significance of differences was assessed by one-way ANOVA with Bonferroni correction for multiple hypothesis testing using StatPlus software (AnalystSoft).

For the pathogenic stress assays, NGM plates were prepared with increased peptone (3.5 g/l; standard is 2.5 g/l). Plates were seeded with 15 µl of a 1:5 dilution of a saturated overnight *P. aeruginosa* PA14 culture. Seeded plates were incubated at 37°C for 24 h, moved to 25°C for another 24 h, then stored at 4°C for a maximum of a week before use.

### RNAi

RNAi by feeding was performed by feeding as previously described (Conte and Mello, 2003). RNAi plates were prepared by supplementing NGM agar with 100 mM ampicillin and 1 mM isopropyl β-D-1-thiogalactopyranoside before pouring. RNAi plates were seeded with *E. coli* HT115 transformed with plasmid that expresses dsRNA targeting *mex-3* or with an empty vector control as previously described (Pagano et al., 2009). Animals were synchronized as L1 larvae, seeded onto RNAi plates, and allowed a minimum of 24 h to feed on MEX-3 RNAi food before imaging or scoring for phenotypes. The effectiveness of the RNAi targeting *mex-3* was confirmed by loss of *gfp::3xflag::mex-3* expression in oocytes and embryos, and by scoring for the strong embryonic lethality phenotype.

### Embryo imaging and phenotyping

Embryos from DG4269, WRM52, WRM75 or WRM77 were recovered from young adults by dissection. Older and terminal embryos were recovered from plates by washing with M9, then repeated centrifugations (956 ***g*** for 1 min) and washes with M9 to isolate embryos from bacteria. Embryos were mounted on 2% agarose pads on glass slides and kept hydrated under the coverslip with 3-5 µl of M9 solution. Images of individual embryos were collected in 20 layers along the *z*-axis using a Zeiss AxioObserver 7 microscope with differential interference contrast and fluorescence imaging optics, a 63× objective and a Zeiss Axiocam 506 mono camera. Fiji software (ImageJ v.2.9.0) was used to create a *z*-projection for each fluorescence channel. For strains expressing multiple fluorescent proteins, all fluorescence channels were merged into a single image for analysis. The genotype and growth condition were documented by one laboratory member, and the state of the embryo (normal, abnormal or dead) was scored by another who was unaware of the embryo identity. The fraction of normal embryos was determined by calculating the number of apparently normal embryos divided by the total number of living embryos.

Young embryos from WRM79, WRM80, WRM81 and WRM82 were recovered and imaged as above. Only embryos older than the 88-cell stage were considered. The total number of cells expressing *Ppie-1::gfp::h2b::nos-2-3′UTR* or *mCherry::pgl-1* was counted. The fraction of normal embryos was determined by calculating the number of embryos with expression in two cells (normal) divided by the total number of embryos scored.

### Embryogenesis movies

Embryos were recovered from young adult hermaphrodites and mounted on 2% agarose pads for imaging as described above. Only embryos that were younger than the 2-cell stage were imaged. DIC and GFP images were collected every 5-7 min. Focus was maintained throughout imaging using a Zeiss Definite Focus unit. Movies were analyzed using Fiji/ImageJ software first by cropping the 16-bit image stacks to 40×60 μm centering the embryo, then measuring the mean GFP::MEX-3 fluorescence across the entire embryo per image in the stack using the ‘Plot Z-axis Profile’ tool. Because embryogenesis cannot be synchronized, time zero was arbitrarily defined as the first frame that showed completed cytokinesis of the first cell division, enabling comparison between multiple embryos. The mean fluorescence intensity was normalized by dividing by the local minimum intensity across all frames of the stack. The average mean fluorescence per time point across at least three embryos was plotted, and the data fit to the following sigmoidal equation using Igor Pro 9.0.2:

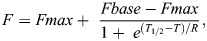
where *F* is the mean GFP intensity across all frames, *Fmax* is the maximal intensity, *Fbase* in the minimum intensity, *T* is the time (in minutes), *T*_1/2_ is the half-time of maximal GFP abundance, and *R* is the rate of loss of steady-state GFP signal, incorporating both synthesis and decay rates. The standard deviation of the fitted parameters was determined from a global fit of all data per genotype and condition.

### Young adult imaging and quantitation

Young adult animals were picked onto a 2% agarose pad on a glass slide in M9 supplemented with levamisole (1 mM) to immobilize them. DIC and GFP images of the germline of intact worms were collected with a Zeiss AxioObserver 7 microscope with a 20× objective and a Zeiss Axiocam 506 mono camera. GFP fluorescence in oocytes was calculated by drawing a line of 30-pixel width across each of the four most proximal oocytes using Fiji software, avoiding the nucleus. The mean fluorescence intensity within the line was measured and the background subtracted for each oocyte. For each genotype and condition, the background-corrected fluorescence intensity for each of the four most proximal oocytes was calculated across at least 12 animals. Differences between the distribution of intensities between oocytes, genotypes and conditions were compared using a one-way ANOVA with Bonferroni correction for multiple hypothesis testing to assess statistical significance.

### RNA-seq

DG4269 and WRM52 animals were cultured at 20°C and 25°C and harvested from unstarved plates with filtered nuclease-free water. Each population was washed at least three times with water prior to RNA isolation using TRIzol, chloroform and isopropanol. Ribosomal RNA from each sample was depleted using a rRNA depletion protocol for *C. elegans* (Duan et al., 2020). Library prep for RNA-seq was performed using the NEBNext Ultra II library prep kit (E7775S) following the manufacturer's protocol. NEBNext Multiplex Oligos for Illumina (Dual Index Primer Set 1; E7600) was used for library indexing. Concentrations for each library was determined using both Qubit and fragment bioanalyzer. Barcoded libraries were sequenced using an Illumina NEXTSeq 1000.

Raw sequencing reads were mapped to the *C. elegans* genome build WBcel135 using the OneStopRNAseq pipeline (Li et al., 2020). Briefly, FastQC v.0.11.5 and MultiQC v.1.6 were used for raw and post-alignment quality control, respectively (https://www.bioinformatics.babraham.ac.uk/projects/fastqc/; Ewels et al., 2016). STAR v.2.7.5a was used to align the reads to the reference genome using WBcel235.90 annotations with default settings except for the following parameters: ‘-Q 20 --minOverlap 1 --fracOverlap 0 -p -B -C’ for paired-end strict-mode analysis (Dobin et al., 2013). Differential expression analysis was performed with DESeq2 v.1.28.1 (Love et al., 2014). Significantly differentially expressed genes were filtered with the criteria FDR<0.05 and absolute log2 fold change (|LFC|)>0.585. The data are available in Table S4. UpSet plot values were calculated by comparing the number of differentially expressed genes for each category that passed both significance and fold change criteria described above using Excel software (Microsoft). The number of upregulated genes was assigned a positive integer and the number of downregulated genes a negative integer to facilitate comparisons on the same axis. The plots were made using Igor Pro 9 software (Wavemetrics).

### Data analysis

All the data and statistical analyses presented in Figs 2–7 are available in Tables S3 and S4.

## Supplementary Material



10.1242/develop.204740_sup1 Supplementary information

Table S3. Numerical data and statistical analysis for Figs 2-6, Fig. S1 and Fig. S2.

Table S4.
