# Supplementary material for: The mex-3 3′ untranslated region is essential for reproduction during temperature stress
Source: Development. 2025 Sep 11;152(17):dev204740. doi: 10.1242/dev.204740 (PMC12710607; doi:10.1242/dev.204740)
Supplement: Supplementary information [file develop-152-204740-s1.pdf]

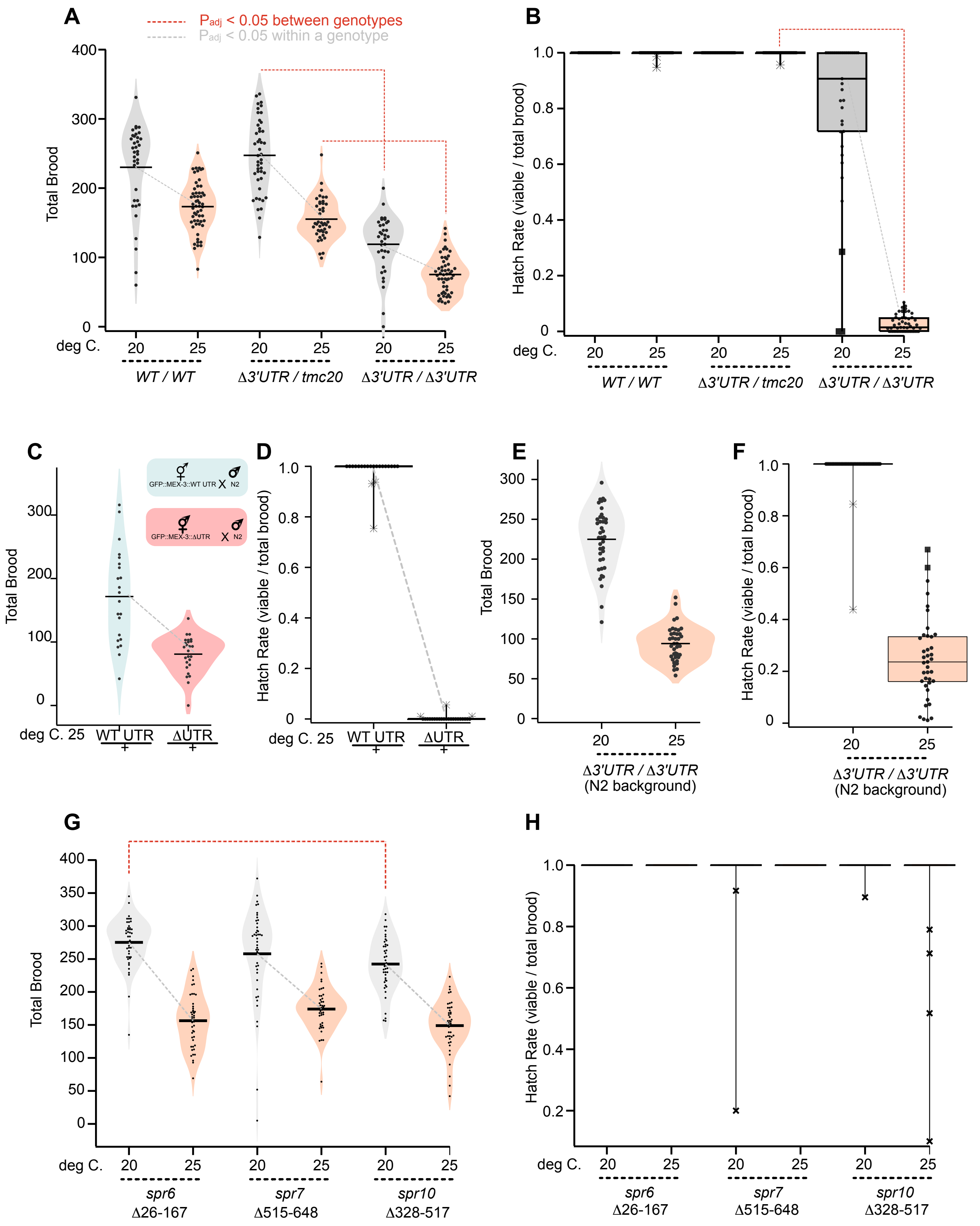
A
P_{adj} < 0.05 between genotypes
P_{adj} < 0.05 within a genotype
Total Brood
deg C.
WT / WT
Δ3'UTR / tmc20
Δ3'UTR / Δ3'UTR
B
Hatch Rate (viable / total brood)
C
GFP::MEX-3::WT UTR X N2
GFP::MEX-3::ΔUTR X N2
deg C. 25
WT UTR
ΔUTR
D
E
Δ3'UTR / Δ3'UTR
(N2 background)
F
G
spr6
Δ26-167
spr7
Δ515-648
spr10
Δ328-517
H

Fig. S1. Additional brood size and hatch rate measurements for a variety of mex-3 alleles. A. Brood size and **B.** hatch rate data for WT/WT (DG4269), Δ3'UTR/tmc20 (WRM89), and Δ3'UTR/ Δ3'UTR (WRM52) hermaphrodites. The data for WT and homozygous Δ3'UTR deletion alleles is reproduced from figure 1. Gray represents 20 degree C. growth, orange represents 25 degree C. growth. Statistical significance was assessed by one-way ANOVA using Bonferroni correction for multiple hypothesis testing. Statistically significant differences between genotypes are indicated by a dashed red line, while significance within a genotype is indicated by a dashed gray line. The thick black bars in panel A indicate the median, the black X marks in panel B represent Tukey outliers. **C.** Brood size and **D.** hatch rate data for the cross progeny of DG4269 hermaphrodites with N2 males compared to WRM52 crossed with N2 males. The data are represented as in panels A and B except blue represents the DG4269 x N2 cross and red indicates the WRM52 x N2 cross. **E.** Brood size and **F.** hatch rate data for *mex-3(spr37)* animals, which harbor a large deletion of the *mex-3* 3'UTR in the N2 background. The data are represented as in panels A and B. **G.** Brood size and **H.** hatch rate data for smaller *mex- 3* deletion alleles. The allele designation and bounds of the deletion are indicated. The data are represented as in panels A and B. All sample sizes and statistical test outcomes are listed in **Table S2**.

Development • Supplementary information

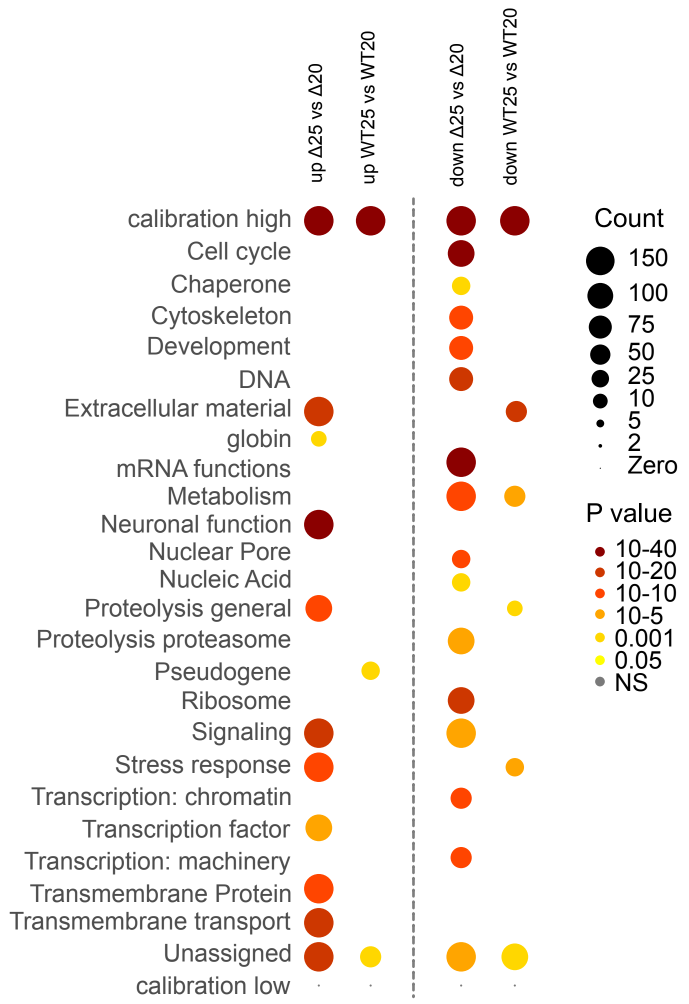

Fig. S2. WORMCAT gene ontology comparison in the *mex-3* 3'UTR deletion mutant and control strain as a function of temperature. The size of the circles indicates the number of genes within a category that are dysregulated. The color of the circle corresponds to the p-value of the enrichment score using default WORMCAT parameters.

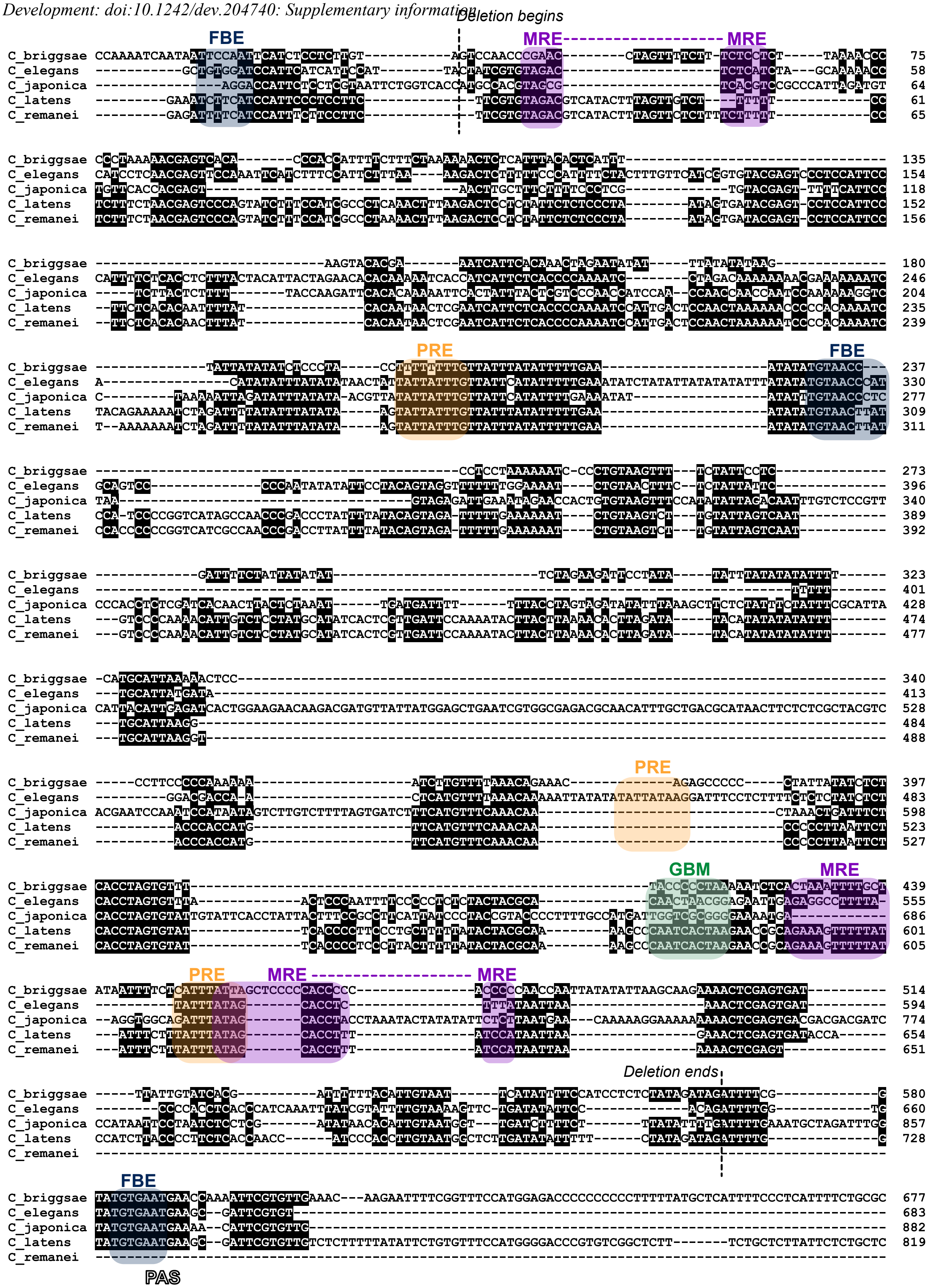
Deletion begins
FBE
MRE
MRE
C_briggsae
C_elegans
C_japonica
C_latens
C_remanei
PRE
FBE
PRE
GBM
MRE
PRE
MRE
MRE
Deletion ends
FBE
PAS

Fig. S3. Additional brood size and hatch rate measurements for a variety of mex-3 alleles. The 3'UTRs of the closest *mex-3* homolog from five *Caenorhabditis* species were recovered from NCBI and aligned using MAFFT within Snapgene software (San Diego, CA) (Katoh et al., 2002). The conserved regions were rendered using pyBoxshade (https://github.com/mdbaron42/pyBoxshade). Motifs for the RBPs were manually annotated on the alignment rendering using colored boxes.

Table S1. Strains used

Strain ID	Genotype
Published Strains	
DG4269	mex-3(tn1753) I<br>*gfp::3xflag::mex-3*<br>(Tsukamoto et al., 2017)
WRM52	mex-3(spr9[*tn1753]) I<br>*gfp::3xflag::mex-3 Δ3´UTR*<br>(Albarqi and Ryder, 2021)
OD1854	ltSi539 II; ltSi507 IV; nre-1(hd20) lin-15B(hd126) X; stIs10389<br>*germ layer reporter*<br>(Wang et al., 2019)
WRM1	sprSi1 II; unc-119(eΔ3) III<br>Ppie-1::gfp::h2b::nos-2 3'UTR<br>(Pagano et al., 2009)
WRM49	mex-3(spr6[*tn1753]) I<br>*gfp::3xflag::mex-3 Δ3´UTR*<br>(Albarqi and Ryder, 2021)
WRM50	mex-3(spr7[*tn1753]) I<br>*gfp::3xflag::mex-3 Δ3´UTR*<br>(Albarqi and Ryder, 2021)
WRM53	mex-3(spr10[*tn1753]) I<br>*gfp::3xflag::mex-3 Δ3´UTR*<br>(Albarqi and Ryder, 2021)

Strains produced for this study	
WRM75	mex-3(spr9[*tn1753]) I; ltSi539 II; ltSi507 IV; nre-1(hd20) lin-15B(hd126) X; stIs10389
WRM77	mex-3(tn1753) I; ltSi539 II; ltSi507 IV; nre-1(hd20) lin-15B(hd126) X; stIs10389
WRM79	mex-3(spr9[*tn1753]) I; sprSi1 II; unc-119(eΔ3) III
WRM80	mex-3(tn1753) I; sprSi1 II; unc-119(eΔ3) III
WRM81	mex-3(spr9[*tn1753]) I; pgl-1(spr20[mCherry::pgl-1] IV
WRM82	mex-3(tn1753) I; pgl-1(spr20[mCherry::pgl-1] IV
WRM89	mex-3(spr9[*tn1753]) / tmC20 [unc-14(tmIs1219) dpy-5(tm9715)] I
WRM113	mex-3(spr37) I

Table S2. Oligonucleotides used

Primer Name	Purpose	Sequence
mex-3_005F	PCR Confirmation of mex-3 3’ UTR genotypes	5’ -CGATCATACTCTCGTGCCGA - 3’
mex-3_007R	PCR Confirmation of mex-3 3’ UTR genotypes	5’ -CTGAAACAATGGGACACCTCAA - 3’
nos-2_H2B_01F	PCR Confirmtion of nos-2 marker	5’ -TACACATGGCATGGATGAACT - 3’
Nos-2_H2B_01R	PCR Confirmation of nos-2 marker	5’ -AAGGCTATGAACGGGTAACTCA - 3’
Linker1_pgl1_HA_F	Generation of pgl-1::mCherry reporter template	5’ - TTAAATATTTATTTCAGTTTCATCCATTTCA CATGTCCGGAGGGAGTGGA - 3’

Development • Supplementary information

Linker2_pgl1_HA_R	Generation of pgl-1::mCherry reporter template	5' - CCACCGAAATCCACAATTTCTCGCTTGTTA GCCTCAGAACCTCCGCCACC - 3'
CD.HC9.YJVG2684.AB	Guide RNA for the generation of spr20	5' – GUUUCAUCCAUUUCACAUGG – 3'
WRM81/82_Sequence _F1	Sequencing and PCR confirmation of spr20	5' – GAGTTTATGCGTTTCAAGGTG – 3'
WRM81/82_Sequence _F2	Sequencing and PCR confirmation of spr20	5' – TCCACAGTTCATGTATGGAAG – 3'
WRM81/82_Sequence _F3	Sequencing and PCR confirmation of spr20	5' – CTATGGGATGGGAAGCTTC – 3'
WRM81/82_Sequence _R1	Sequencing and PCR confirmation of spr20	5' – GCCGGATGTTTAACATAAGC – 3'
WRM81/82_Sequence _R2	Sequencing and PCR confirmation of spr20	5' – CCGTCTTCAGGGTACATTC – 3'
WRM81/82_Sequence _R3	Sequencing and PCR confirmation of spr20	5' – CAATTCATCCATGCCACCT – 3'
WRM113_guide1	Guide RNA for the generation of spr37	5' – GAGAGUCUACACGAUAGUAA – 3'
WRM113_guide2	Guide RNA for the generation of spr37	5' – CCAUUUUCUACUUUGUUCAU– 3'

Development • Supplementary information

WRM113_ssodn	Donor template for generation of spr9 allele in the N2 background	5' – ATGGATAAGCTGTGGATCCATTCATCATTCC ATTAATTTTGGTGTATGTGAATGAAGCGATTC GTGTCGT – 3'

Table S3. Numerical data and statistical analysis for Figs 2-6, Fig. S1 and Fig. S2.

Available for download at
https://journals.biologists.com/dev/article-lookup/doi/10.1242/dev.204740#supplementary-data

Table S4.

Available for download at
https://journals.biologists.com/dev/article-lookup/doi/10.1242/dev.204740#supplementary-data

Development • Supplementary information

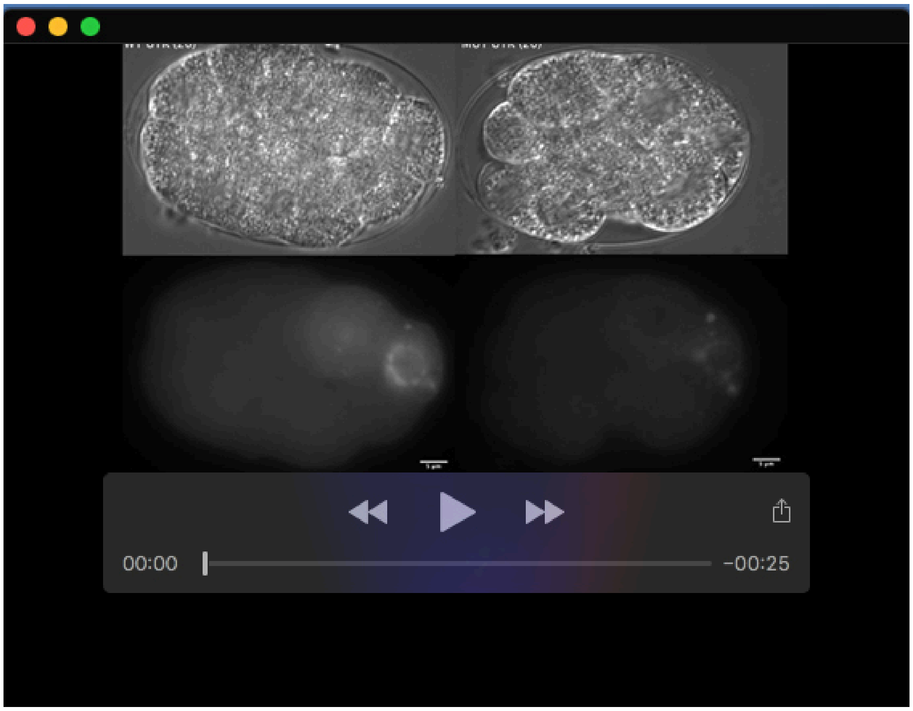

Movie 1. Time course of early embryogenesis comparing representative embryos recovered from the wild-type *gfp:3xflag::mex-3* 3´UTR strain (left) or the *mex-3* Δ3´UTR strain (right) cultured under standard conditions at 20ºC. Frames were collected at a five-minute interval and sped up to a frame rate of 4 frames per second in this movie.

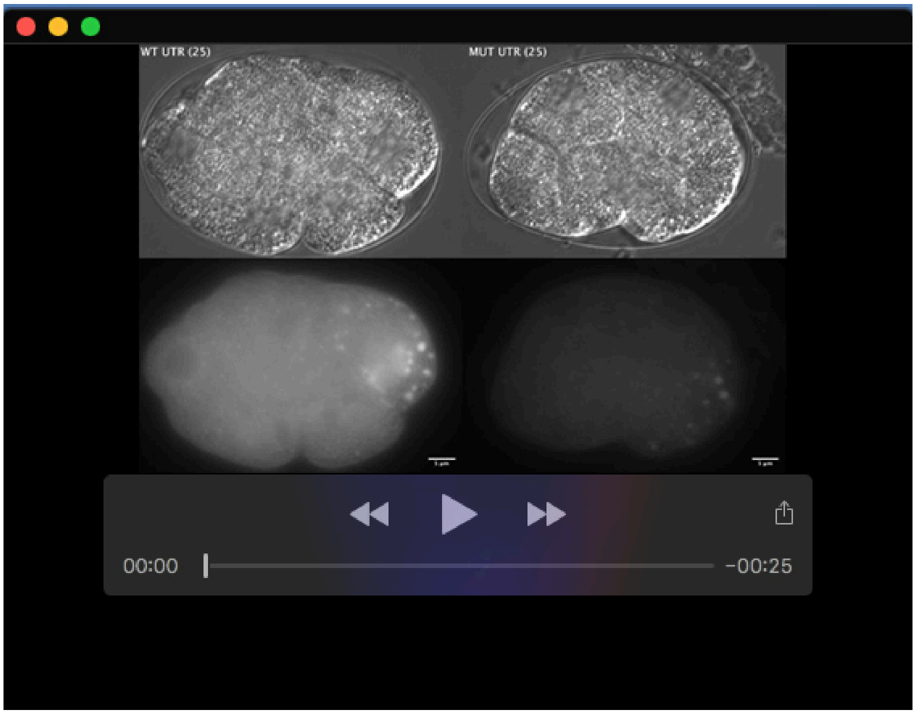

Movie 2. Time course of early embryogenesis comparing representative embryos recovered from the wild-type *gfp:3xflag::mex-3 3*´UTR strain (left) or the *mex-3* Δ3´UTR strain (right) cultured under standard conditions at 25ºC. Frames were collected at a five-minute interval and sped up to a frame rate of 4 frames per second in this movie.

SUPPLEMENTAL REFERENCE

Katoh, K., Misawa, K., Kuma, K. and Miyata, T. (2002). MAFFT: a novel method for rapid multiple sequence alignment based on fast Fourier transform. *Nucleic Acids Res* **30**, 3059-3066.

Development • Supplementary information
