# Supplementary material for: The mex-3 3′ untranslated region is essential for reproduction during temperature stress
Source: Development. 2025 Sep 11;152(17):dev204740. doi: 10.1242/dev.204740 (PMC12710607; doi:10.1242/dev.204740)
Supplement: Supplementary file 1 [file develop-152-204740_review_history.pdf]

The *mex-3* 3' untranslated region is essential for reproduction during temperature stress

Hannah E. Brown, Haik V. Varderesian, Oscar Lam, Sara A. Keane and Sean P. Ryder
DOI: 10.1242/dev.204740

Editor: Swathi Arur

Review timeline
Original submission: 8 April 2024
Editorial decision: 8 May 2024
Resubmission: 25 February 2025
Editorial decision: 11 April 2025
First revision received: 14 July 2025
Accepted: 1 August 2025

Original submission

First decision letter

MS ID#: DEVELOP/2024/202939

MS TITLE: The mex-3 3' untranslated region is essential for reproduction during temperature stress

AUTHORS: Hannah E Brown, Haik V Varderesian, Sara A Keane, and Sean Patrick Ryder
ARTICLE TYPE: Research Article

Dear Dr. Ryder,

I have now received all the referees reports on the above manuscript, and have reached a decision. The referees' comments are appended below, or you can access them online: please go to BenchPress and click on the 'Manuscripts with Decisions' queue in the Author Area.

As you will see from their reports, the referees recognise the potential of your work, however, a major concern is lack of any mechanistic insight into the observed phenotypes observed, for a gene that is otherwise very well characterized in the field. Given the nature of these concerns, I am afraid I have little choice other than to reject the paper at this stage.

However, having evaluated the paper, I do recognise the potential importance of this work. I would therefore be prepared to consider as a new submission an extension of this study that contains new experiments, data and discussions and that address the underlying mechanism that may be driving the UTR dependent phenotypes of mex-3. The work required goes beyond a standard revision of the paper. Please bear in mind that the referees (who may be different from the present reviewers) will assess the novelty of your work in the context of all previous publications, including those published between now and the time of resubmission.

Reviewer 1 Advance Summary and Potential Significance to Field:

In “The mex-3 3' untranslated region is essential for reproduction during temperature stress”, Brown and colleagues report a remarkable finding that a mex-3 3’UTR deletion strain originally reported in Albarqi et. al. has a highly penetrant temperature sensitive embryonic lethality

© 2024. Published by The Company of Biologists under the terms of the Creative Commons Attribution License (https://creativecommons.org/licenses/by/4.0/).

phenotype. This result is surprising. Loss of MEX-3 results in highly-penetrant embryonic defects. Yet, the 3' UTR is sufficient to pattern MEX-3 expression yet is dispensable under standard growth conditions. The authors conclude that the mex-3 3´UTR serves a critical role as a buffer for thermal environmental stress. This work greatly expands our understanding of a very well characterized model – the mex-3 3´UTR – and does so with exquisite precision afforded by bespoke genome editing at endogenous loci. This work is interesting, rigorous, and well-articulated. I have several minor suggestions intended to improve an already strong manuscript.

Reviewer 1 Comments for the Author:
I am intrigued by the proposed models for mex-3 function. I wonder if it would be worthwhile to test how a translation inhibitor would impact the repressive circuit (something like homoharringtonine would probably work because of the conservation of the target but that won't trigger elongation stress like Anisomycin)? If the idea is that Mex-3 overexpression drives exaggerated repression of cell fate specification programs at the level of translation, one would expect that small molecules that antagonize the process would rescue the 3'UTR deletion TS phenotype. If instead, RNA structure or protein folding are the key mechanism, this might not be impacted by a translational inhibitor. There are of course other interpretations but these explanations are parsimonious. To confirm the impact of an inhibitor on translation, the authors could validate it with a simple puro pulse chase if desired (Fig. 6 - https://www.ncbi.nlm.nih.gov/pmc/articles/PMC4245946/).

Typos:
Page 2 RNA-binding
Page 3 mutants as compared to…
Page 11 suggest trans-acting factors
Legend figure 4 – phenotypes of
Legend figure 4 - last sentence is odd
Legend figure 7 – A hypothetical model…
Citations – I am amused and confused that paper 3 has no authors. Graduation from that lab must be challenging.

***** Reviewer 2 Advance Summary and Potential Significance to Field:

The manuscript "The mex-3 3´ untranslated region is essential for reproduction during temperature stress" by Brown et al expands upon the previous studies into the roles of an essential RNA binding protein, MEX-3, during germline and embryo development. MEX-3 is a conserved protein with critical functions and is an excellent model to study RBP function in the germline and as a maternally loaded product in early embryogenesis. Given the conservation of these mechanisms, studies into the roles of maternally loaded products in early embryonic patterning and cell fate specification are invaluable for understanding of development across clades. Specifically, this well-written manuscript describes the observation that mex-3 3' UTR, while largely dispensable at normal developmental temperatures, becomes essential during thermal stress. The study also compares a variety of stresses and carefully describes the distinct effects of the mex- 3 3' UTR deletion on the germline and early embryogenesis. Since this study addresses the potential molecular mechanisms underlying developmental robustness under variable conditions, it is likely to be of interest to a broad audience.

Reviewer 2 Comments for the Author:
The use of single and double asterisks to indicate significance, especially in Figure 2, is confusing. It's not immediately apparent that three asterisks are just a combination of one plus two and not another designation of significance. In addition, I found myself constantly checking whether * vs. ** meant within the genotype or across. It was cumbersome. While I sympathize that lines indicating comparisons and statistical significance make the figures busy, I think that's preferable to the difficulty the reader has in making the comparisons in the first place.

Figure 4 should include RNAi data. Because the manuscript compares the effects of RNAi (as a strong loss of function phenotype) vs. UTR deletion, it's helpful to have the data quantified for comparison to/in panel C. Figure 4 lacks white arrows mentioned in A pointing to the legend-described phenotypes.

© 2024. Published by The Company of Biologists under the terms of the Creative Commons Attribution License (https://creativecommons.org/licenses/by/4.0/).

Figure 7C relates to the discussion on potential differences in mex-3 UTR folding. It seems that the discussion would very much benefit from a comparison of the two folds at both 20C and 25C. For example, while, of course, unlikely, it could be that the 3' UTR predicted fold is identical at both temperatures, which would make the discussion of the temperature-dependent differences totally moot. In addition, no information was provided on the folding parameters in Materials and Methods and this information (such as temperature) is critical (for example, the default temperature for RNAfold is 37C and thus wildly inappropriate for C. elegans RNA folding predictions).

The abstract statement that mex-3 3' UTR "is dispensable for fertility" is slightly misleading. The previous manuscript does show some effect of the 3'UTR deletion mutant on fertility. Perhaps "largely dispensable" or some such version would be best.

Methods and Materials has a placeholder instead of final levamisole concentration.

End of Introduction: typo, "reduction in total and viable brood"

***** Reviewer 3 Advance Summary and Potential Significance to Field:

MEX-5 is an RNA-binding protein required for embryonic development in C. elegans. Like other maternally- provided proteins, MEX-3 levels peak in mature oocytes and decay in embryos in a manner dependent on the MEX-3 3'UTR. This paper reports on the phenotype of a mex-3 mutant missing a portion of the 3'UTR. The UTR-deletion mutant causes premature low MEX-3 expression throughout germline development with no increase in maturing oocytes leading to reduced levels in embryos. The UTR-deletion mutant exhibits reduced embryo viability, most dramatically at low and high temperatures (compared to 20oC standard temp). Consistent with reduced protein levels in embryos, characterization of the embryonic phenotype suggests that the mutant behaves like a partial loss of function. The UTR-deletion mutant also exhibits reduced brood size and delayed rescue upon shift from high to permissive temperature, suggestive of a defect intrinsic to the germline but this was not explored further.

The data presented are thorough and well presented, but unfortunately do not provide any mechanistic insights into either the cause for the embryonic lethality nor the origin of the temperature sensitivity. The UTR-deletion removes several binding sites for known regulators but the authors do not investigate smaller deletions that might shed light as to the origin of the phenotypes described. It is not clear whether the phenotypes are due to reduced expression in embryos or premature expression in the germline or a combination of both.

Reviewer 3 Comments for the Author:
Specific comments:

-Fig. 3 suggest that MEX-3 levels are reduced in embryos in the mutant, but this was not quantified? Do the curves show in D represent one embryo of each genotype? Is it reproducible that MEX-3 levels are reduced at 25oC even in wild-type embryos as suggested in Panel D?
-Fig. 5C – data for mex-3(RNAi) should be included here?
-Does MEX-3 null mutants also display temperature sensitive variations in their phenotypes?
-Fig. 7 is a speculative model that is not tested or explored by the data presented in the paper?

First revision

Author's response to the reviewer's comments

Point-by-Point Response:

We thank all the reviewers for their insightful comments and helpful suggestions. In this revised manuscript we have addressed all the concerns and corrected all the mistakes / typos that were

© 2024. Published by The Company of Biologists under the terms of the Creative Commons Attribution License (https://creativecommons.org/licenses/by/4.0/).

identified in the prior round of review. In addition, we have added new data that expands the scope of the work. The new data include characterization of three additional *mex-3* 3'UTR mutants (Supplemental Figure 1) as well as a comprehensive RNA-seq analysis performed at both permissive and restrictive temperature for the *mex-3(spr9)* mutant compared to a genotype background matched control (new Figure 7 and Supplemental Figure 2). The RNA-seq data reveal a surprising impact of the mutation on heat shock response gene expression, speaking to the mechanism of the temperature sensitive embryonic lethality phenotype. Specifically, the *mex-3(spr9)* mutation causes decreased expression of more than half of all known heat shock response genes, explaining why this mutant is so sensitive to temperature, and suggesting a new role for MEX-3 in establishing the baseline of this response. We have edited the models that we presented in our discussion section to reflect the new findings.

Reviewer #1

This work greatly expands our understanding of a very well characterized model – the mex-3 3´UTR – and does so with exquisite precision afforded by bespoke genome editing at endogenous loci. This work is interesting, rigorous, and well-articulated. I have several minor suggestions intended to improve an already strong manuscript.

We thank the reviewer for their interest and for the favorable review of our work.

If the idea is that Mex-3 overexpression drives exaggerated repression of cell fate specification programs at the level of translation, one would expect that small molecules that antagonize the process would rescue the 3'UTR deletion TS phenotype.

The failure of the activation circuit, rather than the repressive circuit, appears to be responsible for the temperature sensitive embryonic lethality phenotype. MEX-3 is ectopically expressed in the germline at both permissive and restrictive temperatures. Elevated temperature causes a specific reduction of MEX-3 in the most proximal oocyte and in embryos, suggesting that the embryonic lethality phenotype is due to less MEX-3 in embryos, rather than more MEX-3 in the germline. Consistent with this idea, some but not all features of the terminal embryos resemble a mex-3 null phenotype.

Typos:
Page 2 RNA-binding
Page 3 mutants as compared to…
Page 11 suggest trans-acting factors
Legend figure 4 - phenotypes of
Legend figure 4 - last sentence is odd
Legend figure 7 – A hypothetical model…

We thank the reviewer for identifying our typographical errors and points of non-clarity. We have made all of the suggested changes.

Reviewer #2

Given the conservation of these mechanisms, studies into the roles of maternally loaded products in early embryonic patterning and cell fate specification are invaluable for understanding of development across clades. Specifically, this well-written manuscript describes the observation that mex-3 3' UTR, while largely dispensable at normal developmental temperatures, becomes essential during thermal stress. The study also compares a variety of stresses and carefully describes the distinct effects of the mex-3 3' UTR deletion on the germline and early embryogenesis. Since this study addresses the potential molecular mechanisms underlying developmental robustness under variable conditions, it is likely to be of interest to a broad audience.

We thank the reviewer for their thoughtful overview of our work and their assessment of its potential impact.

© 2024. Published by The Company of Biologists under the terms of the Creative Commons Attribution License (https://creativecommons.org/licenses/by/4.0/).

The use of single and double asterisks to indicate significance, especially in Figure 2, is confusing. It's not immediately apparent that three asterisks are just a combination of one plus two and not another designation of significance. In addition, I found myself constantly checking whether * vs. ** meant within the genotype or across. It was cumbersome. While I sympathize that lines indicating comparisons and statistical significance make the figures busy, I think that's preferable to the difficulty the reader has in making the comparisons in the first place.

We have redesigned the figures to make statistical significance comparisons both within and between genotypes more obvious. The changes can be seen as dashed lines in Figures 2 and 3 as well as Supplemental Figure 1.

Figure 4 should include RNAi data. Because the manuscript compares the effects of RNAi (as a strong loss of function phenotype) vs. UTR deletion, it's helpful to have the data quantified for comparison to/in panel C.

We completely agree and apologize for our oversight. The RNAi data is now quantified alongside the mutant data throughout the manuscript. Please see Figures 4-6.

Figure 7C relates to the discussion on potential differences in mex-3 UTR folding. It seems that the discussion would very much benefit from a comparison of the two folds at both 20C and 25C. For example, while, of course, unlikely, it could be that the 3' UTR predicted fold is identical at both temperatures, which would make the discussion of the temperature-dependent differences totally moot. In addition, no information was provided on the folding parameters in Materials and Methods and this information (such as temperature) is critical (for example, the default temperature for RNAfold is 37C and thus wildly inappropriate for C. elegans RNA folding predictions).

We agree with the reviewer's assessment and repeated the RNAfold analysis as a function of temperature (20-25 deg. C). We did not observe any potential temperature dependent variation in the predicted secondary structure. In light of new data from RNA sequencing, we no longer favor this model to explain the basis of the temperature sensitive phenotype and have removed it from the manuscript. Our new models, based on the RNA sequencing data presented in Figure 7, are found in the Discussion on page 13.

The abstract statement that mex-3 3' UTR "is dispensable for fertility" is slightly misleading. The previous manuscript does show some effect of the 3'UTR deletion mutant on fertility. Perhaps "largely dispensable" or some such version would be best.

We have made the suggested change, and corrected the additional typographical errors found by the reviewer.

Reviewer #3

The data presented are thorough and well presented, but unfortunately do not provide any mechanistic insights into either the cause for the embryonic lethality nor the origin of the temperature sensitivity.

We agree with the reviewer. These are in fact limitations of our previous manuscript. In the new version, we have addressed both with new experiments (presented in Figure 7 and Supplementary Figures 1 and 2). The new data evaluate the impact of the mutation on gene expression at both permissive and restrictive temperature. They reveal a dramatic effect on genes in the heat shock response pathway. Specifically, more than half of the heat shock response genes are downregulated in the mutant at permissive temperature compared to a genotype matched control, indicating a role for MEX-3 in establishing the baseline response to temperature. This explains why this mutant—in the noncoding region of a maternal mRNA—displays such a dramatic temperature sensitive phenotype. Upon increasing temperature, the identity and number of gene expression changes observed in the mutant is strikingly different compared to the control (see Figure 7), with thousands of changes (both up and down) unique to the mutant at elevated temperature. The new data provide a plausible mechanism for the basis

© 2024. Published by The Company of Biologists under the terms of the Creative Commons Attribution License (https://creativecommons.org/licenses/by/4.0/).

of the temperature sensitive phenotype, and for the mechanisms of embryonic lethality.

The UTR-deletion removes several binding sites for known regulators but the authors do not investigate smaller deletions that might shed light as to the origin of the phenotypes described. It is not clear whether the phenotypes are due to reduced expression in embryos or premature expression in the germline or a combination of both.

In the new manuscript, we evaluate three additional mex-3 3'UTR alleles that are shorter than the large deletion allele presented in the previous manuscript. The new data is presented in Supplemental Figure 1. None of the shorter mutations shows the same strong temperature sensitive embryonic lethality phenotype as the near full-length deletion, suggesting that binding sites scattered throughout the UTR contribute to the strong dysregulation and temperature sensitivity phenotypes that we observed.

Our data support a model where the temperature sensitive embryonic lethality phenotype is caused at least in part by the reduced expression in embryos. Three lines of evidence support this model. First, ectopic expression in the germline is observed at permissive temperature, but the embryonic lethality phenotype only manifests strongly at the restrictive temperature (Figure 2B). In contrast to germline expression, the reduction in oocytes and embryos is temperature dependent. Second, the increase in mesoderm expression observed at restrictive temperature in embryos resembles the mex-3 RNAi phenotype (Figure 5A,B). Finally, the increased number of germline progenitor cells observed in embryos at restrictive temperature is similar to the mex-3 RNAi phenotype (Figure 6A,B). The latter two results are less severe in the 3'UTR mutant compared to the RNAi phenotype, suggesting that the 3'UTR deletion is a temperature sensitive hypomorph. Having said that, we cannot rule out the possibility that the combination of overexpression in the germline and reduced expression in the embryo is important for the phenotype. Indeed, our new RNA-seq data show strong gene expression changes between the mutant and control strains at both temperatures.

Fig. 3 suggest that MEX-3 levels are reduced in embryos in the mutant, but this was not quantified? Do the curves show in D represent one embryo of each genotype? Is it reproducible that MEX-3 levels are reduced at 25oC even in wild-type embryos as suggested in Panel D?

The reduction of MEX-3 abundance in embryos is quantified in Figure 3 panel D. The data presented in Figure 3D for each time point represent the average and standard deviation for an N of two to five movies for each genotype under each condition. The data are fit to a sigmoidal decay equation to determine the initial amount and the half-life for each protein. We agree that this wasn't clear in our previous manuscript and have edited the legend to Figure 3 and the materials and methods section to clarify what Figure 3D represents.

Fig. 5C – data for mex-3(RNAi) should be included here?

We apologize for our oversight and have added quantitation of the RNAi data to figures 4-5.

Does MEX-3 null mutants also display temperature sensitive variations in their phenotypes?

The phenotype of a mex-3 null is 100% embryonic lethality at 20 degrees. This strain must be propagated as a balanced heterozygote as a result. The phenotype of the mex-3 null is more severe than the mex-3(spr9) mutation presented in our work. The embryos fail prior to morphogenesis. We have not observed stronger phenotypes (for example sterility or arrest at in earlier embryonic stages) in trials with mex-3 null animals.

Fig. 7 is a speculative model that is not tested or explored by the data presented in the paper?

In the previous manuscript, Fig. 7 did include speculative models to explain the temperature mutant phenotype. We have collected new data (presented in a new Figure 7) that speaks to a different mechanism of the temperature sensitivity phenotype. As such, we have removed the previous version of Figure 7 from the manuscript as well as the discussion of these hypothetical models.

© 2024. Published by The Company of Biologists under the terms of the Creative Commons Attribution License (https://creativecommons.org/licenses/by/4.0/).

Resubmission

First decision letter

MS ID#: dev.204740

MS TITLE: The mex-3 3' untranslated region is essential for reproduction during temperature stress

AUTHORS: Hannah E Brown, Haik V Varderesian, Sara A Keane, Sean Patrick Ryder

Dear Dr Ryder,

I have now received all the referees' reports on the above manuscript, and have reached a decision. The referees' comments are appended below, or you can access them online: please go to:

As you will see, the referees express considerable interest in your work, but have some significant recommendations that need to be addressed, which will require a substantial revision of your manuscript before we can consider publication. If you are able to revise the manuscript along the lines suggested, which involve further experiments, I will be happy receive a revised version of the manuscript. Your revised paper will be re-reviewed by one or more of the original referees, and acceptance of your manuscript will depend on your addressing satisfactorily the reviewers' major concerns. Please also note that Development will normally permit only one round of major revision.

Please attend to all of the reviewers' comments and ensure that you clearly highlight all changes made in the revised manuscript. Please avoid using 'Tracked changes' in Word files as these are lost in PDF conversion. I should be grateful if you would also provide a point-by-point response detailing how you have dealt with the points raised by the reviewers in the 'Response to Reviewers' box. If you do not agree with any of their criticisms or suggestions please explain clearly why this is so.

Reviewer 1

SUMMARY OF THE ADVANCE MADE IN THIS PAPER AND ITS POTENTIAL SIGNIFICANCE TO THE FIELD

MEX-3 is a conserved RNA-binding protein required for oocyte maturation and embryogenesis in the C. elegans model organism. The transcript is provided maternally and heavily regulated in its 3'UTR through other conserved RNA-binding proteins. Removing a majority of this 3'UTR affects protein expression but the mutant is surprisingly dispensible overall for fertility. Here Brown, et al. further investigate the molecular and biological phenotypes of this mex-3 3'UTR mutant. The manuscript shows evidence that the mex-3 3'UTR mutant has sensitivity to temperature stress in brood size and hatching rate. Protein expression levels are affected at different temperatures in oocytes and in early embryos, and mex-3 3'UTR mutants show defects in animal development at the starting as early as the comma stage. The 3'UTR mutant showed low penetrance of a progenitor cell expansion phenotype associated with mex-3 null mutants. RNA expression of wild type and mutant animals at different temperatures revealed differences between strains, but most notably decreased expression of heat-shock associated proteins in mutant animals.

This will work uses mex-3 as a model maternal transcript to probe the necessity of regulatory elements to environmental stressors and animal development. The manuscript was very well written and incredibly cited. It has (sadly) been a while since I have read such an interesting introduction with valuable references. Based upon the previous reviewers' criticisms, there is a very high likelihood that I would have made similar suggestions in the first draft. The RNA-seq data touches upon and supportive of the mechanism suggested by the manuscript. My comments are very minor. This work will be of interest to developmental and RNA biologists.

SUGGESTIONS TO AUTHORS

© 2024. Published by The Company of Biologists under the terms of the Creative Commons Attribution License (https://creativecommons.org/licenses/by/4.0/).

MINOR

1. Abstract (and similar sentence at the end of the introduction): "We propose that the primary role of the mex-3 3´UTR is to buffer MEX-3 expression during fluctuating temperature, ensuring the robustness of oocyte maturation and embryogenesis." I understand the manuscript's intentions with this sentence, but surely the primary "role" of any 3'UTR is for adequate translation, as complete removal of the UTR will most likely prevent any protein expression. Recommend changing the word, "role," to "design" or something similar.
2. Figure 8 did not highlight anything about mex-3's role in managing temperature fluctuations for embryogenesis. The information felt tangential to the main crux of the manuscript.
3. The Methods section should briefly describe the UpSet plot generation presented in Figure 7.

Reviewer 2

SUMMARY OF THE ADVANCE MADE IN THIS PAPER AND ITS POTENTIAL SIGNIFICANCE TO THE FIELD

MEX-3 is an essential C. elegans RNA-binding protein with a dynamic expression pattern in the germline: MEX-3 is expression is low in the distal gonad, increases as oocytes mature and is subsequently degraded around the activation of the zygotic genome in the early embryo. Previous work from the Ryder group showed that deletion of the GFP::MEX-3 3'UTR (GFP::MEX-3 delta 3'UTR) results in uniform expression in the gonad. GFP::MEX-3(delta 3'UTR) animals were found to be reasonably healthy at 20C, with only a moderate decrease in fecundity (Albarqi 2021).
Brown et al extend this previous work to show that GFP::MEX-3(delta 3'UTR) animals exhibit significant fecundity and embryonic lethality defects at the stressful culture temperature of 25C. GFP::MEX-3(delta 3'UTR) expression levels are reduced relative to wildtype GFP::MEX-3 at 20C or 25C. The embryonic patterning defects of GFP::MEX-3(delta 3'UTR) embryos are less severe than mex-3(RNAi) embryos, indicating GFP::MEX-3(delta 3'UTR) embryos retain some MEX-3 function. Transcriptomics analysis identified many hundreds of genes whose expression is altered in GFP::MEX-3(delta 3'UTR) animals. Roughly half of previously identified heat stress responsive genes are expressed at lower levels in GFP::MEX-3(delta 3'UTR) relative to GFP::MEX-3 animals at 20C, which the authors suggest may account for the temperature sensitive fertility defects of these animals at 25C.
It seems likely that the phenotypes described result from decreased expression of GFP::MEX-3(delta 3'UTR), although this was not directly tested. The gene expression changes in GFP::MEX-3(delta 3'UTR) provide an intriguing correlation with the temperature sensitive fertility defects. However, the importance of these gene expression changes are not tested functionally (this would be a difficult experiment). Additionally, how deletion of the MEX-3 3'UTR leads to changes in these genes remains unexplored. While the data are presented clearly and are reasonably interpreted, the new insight into MEX-3 regulation or function is limited. The experiments required to extend the conceptual advances of this study are long-term and somewhat open-ended.

SUGGESTIONS TO AUTHORS

My understanding is that these experiments are carried out exclusively in the GFP::MEX-3 background. To rule out potential synthetic interactions between the GFP and the 3'UTR deletion, I suggest charactering the 3'UTR deletion in the untagged MEX-3 background.
Since PAL-1 is a well characterized MEX-3 target, it would be interesting to stain for PAL-1 in GFP::MEX-3(delta 3'UTR) embryos. Is it derepressed in the anterior? How does its expression in GFP::MEX-3(delta 3'UTR) compare to embryos partially depleted of MEX-3?
While this is beyond the scope of this paper, I wonder whether a GFP::MEX-3(delta 3'UTR) suppressor screen might help uncover the basis for the temperature sensitive fertility defects.
The change in GFP::MEX-3 expression in the embryo reflects the balance of GFP::MEX-3 degradation and new GFP::MEX-3 synthesis/maturation. Therefore, I would not estimate protein half-life from the decay curves in Figure 3D. The apparent decrease in the half-life of GFP::MEX-3(delta 3'UTR) could be due to decreased rates of synthesis/maturation.
How does the expression of heat stress responsive genes compare between GFP::MEX-3 and GFP::MEX-3(delta 3'UTR) animals at 25C? I appreciate that the lethality etc in the GFP::MEX-3(delta 3'UTR) animals might make this comparison challenging, but perhaps a comparison following acute heat stress might be interpretable.

© 2024. Published by The Company of Biologists under the terms of the Creative Commons Attribution License (https://creativecommons.org/licenses/by/4.0/).

Reviewer 3

In their article, "The mex-3 3' untranslated region is essential for reproduction during temperature stress," Brown and colleagues report a remarkable finding: a mex-3 3'UTR deletion strain originally described by Albarqi et al. exhibits a highly penetrant, temperature-sensitive embryonic lethality phenotype. This result is surprising. While loss of MEX-3 results in highly penetrant embryonic defects, the 3'UTR which is sufficient to pattern MEX-3 expression is dispensable under standard growth conditions. The authors conclude that the mex-3 3´UTR plays a critical role in buffering against thermal environmental stress, osmotic stress, and pathogenic stress. This work greatly expands our understanding of a well-characterized model the mex-3 3´UTR and does so with exquisite precision made possible by bespoke genome editing at endogenous loci. Of note in the current manuscript are new data that illuminate how the mutation impacts gene expression at restrictive and permissive temperatures. Notably, half of the heat shock responsive genes are reduced in the mutant suggesting that MEX-3 defines a latent network of temperature sensitivity. This is the key missing link between the phenotypic observations previously reported and regulatory disruptions triggered by loss of the MEX-3 UTR. This study is insightful, methodologically rigorous, and well-articulated.

This work prompted several questions that I have for the authors:

1. Related to -trans acting factors and their regulation of mex-3, have the authors examined conservation of these elements across nematodes?
2. Could activation of Mex-3 result from de-repression rather than selective translation?
3. The ribosome connection the authors report is intriguing. Elongation rates have been linked to thermogenesis in mammals - PMID: 31417118, PMID: 25353634, PMID: 30890561. In addition to detection of ribosomal proteins, do the authors see any misregulation of these factors? That would be a fascinating parallel.
4. How do differences in mRNA abundance that encode heat shock proteins explain the phenotype with the pseudomonas pathogen? Can the authors speculate on that facet of the story?

There is clearly more work to be done with this line of investigation but this paper makes a substantial and fascinating contribution to the literature that will be of broad interest to the community.

First revision

Author response to reviewers' comments

Point-by-Point Response:

We thank all the reviewers for their insightful comments and helpful suggestions concerning our research. In this revision, we have addressed the concerns and corrected the mistakes / typos that were identified in the prior round of review. As a result, the manuscript describes additional experiments and new data that expand the scope of the work. The new data include characterization of a new *mex-3* 3'UTR deletion mutant made in the N2 background, additional studies to assess the impact of the mutation in heterozygotes, including generation of balanced strains and evaluating N2 cross progeny (Supplemental Figure 1). We have also performed additional analyses of the RNA-sequencing data collected at elevated temperature (Figure 7), as well as an analysis of conservation of candidate cis-regulatory motifs in the *mex-3* 3'UTR (Supplemental Figure 3). The new data are consistent with the models proposed in the previous version and add rigor to the manuscript. Again, we are grateful to the reviewers for their thoughtful comments and suggestions. We have done our best to address each one in the revision. Please find detailed responses below.

Reviewer #1

© 2024. Published by The Company of Biologists under the terms of the Creative Commons Attribution License (https://creativecommons.org/licenses/by/4.0/).

The manuscript was very well written and incredibly cited. It has (sadly) been a while since I have read such an interesting introduction with valuable references. Based upon the previous reviewers' criticisms, there is a very high likelihood that I would have made similar suggestions in the first draft. The RNA-seq data touches upon and supportive of the mechanism suggested by the manuscript. My comments are very minor. This work will be of interest to developmental and RNA biologists.

We thank the reviewer for their interest and for the favorable review of our work, and for the compliment. We try hard to be respectful of the research and researchers who came before us.

Abstract (and similar sentence at the end of the introduction): "We propose that the primary role of the mex-3 3´UTR is to buffer MEX-3 expression during fluctuating temperature, ensuring the robustness of oocyte maturation and embryogenesis." I understand the manuscript's intentions with this sentence, but surely the primary "role" of any 3'UTR is for adequate translation, as complete removal of the UTR will most likely prevent any protein expression. Recommend changing the word, "role," to "design" or something similar.”

We understand and appreciate the reviewer’s point. We have edited the text in both places to replace “primary role” with “major function”, which hopefully better captures our intent without going too far.

Figure 8 did not highlight anything about mex-3's role in managing temperature fluctuations for embryogenesis. The information felt tangential to the main crux of the manuscript.

We have removed figure 8, we agree that it is off topic and too speculative given the data presented here.

3. The Methods section should briefly describe the UpSet plot generation presented in Figure 7.

A section describing the generation of the UpSet plots was added to the materials and methods on page 29.

Reviewer #2

While the data are presented clearly and are reasonably interpreted, the new insight into MEX-3 regulation or function is limited. The experiments required to extend the conceptual advances of this study are long-term and somewhat open-ended.

We thank the reviewer for their thoughtful overview of our work and their assessment of its impact. We have done our best to improve the work by performing most of the experiments that were suggested. While some could not be completed within the window allotted to submit a revised manuscript, we are continuing to work on this project, and some of the experiments suggested by the reviewer that we could not complete will surely be included follow up work.

My understanding is that these experiments are carried out exclusively in the GFP::MEX-3 background. To rule out potential synthetic interactions between the GFP and the 3'UTR deletion, I suggest charactering the 3'UTR deletion in the untagged MEX-3 background.

This is a fair point, and we agree that it is important to address this potential caveat. We remade the largest mex-3 3'UTR deletion in the N2 wild type background. The new data is shown in supplementary figure 1E and F. We observe the same temperature dependent embryonic lethality phenotype in the N2 background as in the GFP-tagged strain, but the penetrance is not as high (~75% lethality compared to ~97% lethality), suggesting that the GFP tag has some influence on the phenotype magnitude.

While this is beyond the scope of this paper, I wonder whether a GFP::MEX-3(delta 3'UTR) suppressor screen might help uncover the basis for the temperature sensitive fertility defects.

© 2024. Published by The Company of Biologists under the terms of the Creative Commons Attribution License (https://creativecommons.org/licenses/by/4.0/).

We thought this was an excellent suggestion and put considerable effort into trying to identify a suppressor of the 3'UTR deletion mutant. We crossed the mex-3(spr9) allele into a vulvaless lin-2(e1309) mutant. Homozygotes of both alleles form bags of worms at permissive temperature and bags of dead embryos at restrictive temperature. We performed an ENU suppressor screen to try to find animals that would yield bags of live worms at 25 degrees, but did not identify any suppressing alleles. The RNA-seq data may provide a reason. If the embryonic lethality is due to the cumulative effects of dysregulation of hundreds of genes, then a single suppressing mutant may not arise. However, it's possible that we haven't yet saturated the screen, and that rare alleles could be identified through additional screening. We do intend to continue down this path, although we have decided not to describe this effort in our present revision due to the negative outcome thus far.

Since PAL-1 is a well characterized MEX-3 target, it would be interesting to stain for PAL-1 in GFP::MEX-3(delta 3'UTR) embryos. Is it derepressed in the anterior? How does its expression in GFP::MEX-3(delta 3'UTR) compare to embryos partially depleted of MEX-3?

This is a good suggestion. Both pal-1 and and nos-2 mRNA are well characterized targets of MEX-3. We characterized nos-2 in our manuscript (Figure 6), but we have not assessed the impact on PAL-1 expression. We intend to follow up with a thorough characterization of PAL-1 and additional candidate targets that emerged from our RNA-seq data sets in a future study.

The change in GFP::MEX-3 expression in the embryo reflects the balance of GFP::MEX-3 degradation and new GFP::MEX-3 synthesis/maturation. Therefore, I would not estimate protein half-life from the decay curves in Figure 3D. The apparent decrease in the half-life of GFP::MEX-3(delta 3'UTR) could be due to decreased rates of synthesis/maturation.

The reviewer is absolutely correct. The steady state GFP abundance reflects both synthesis and decay rates. We have edited the language in the results, methods, and figure legends to make this fact clear (p. 10, p. 28)

How does the expression of heat stress responsive genes compare between GFP::MEX-3 and GFP::MEX-3(delta 3'UTR) animals at 25C? I appreciate that the lethality etc in the GFP::MEX-3(delta 3'UTR) animals might make this comparison challenging, but perhaps a comparison following acute heat stress might be interpretable.

We have performed the suggested analysis with the 25 deg. C RNA seq data and added it to figure 7, panel F.

Reviewer #3

This is the key missing link between the phenotypic observations previously reported and regulatory disruptions triggered by loss of the MEX-3 UTR. This study is insightful, methodologically rigorous, and well-articulated.

We thank the reviewer for their evaluation of our works impact and rigor, and for the compliment on our presentation.

Related to -trans acting factors and their regulation of mex-3, have the authors examined conservation of these elements across nematodes?

This is a great suggestion. We aligned and compared the mex-3 3'UTRs from five Caenorhabditis species where the 3'UTR has been annotated. The alignment is provided in Supplemental Figure 3 and described on page 20. The alignment shows that some of the predicted binding sites are conserved, while others are not. There are also several regions of extensive conservation where the predicted binding sites have been mapped. Identifying the regions of the UTR that govern different aspects of the observed phenotypes, and which trans-acting regulators are involved, will be a major direction of my labs' research over the next few years.

Could activation of Mex-3 result from de-repression rather than selective translation?

© 2024. Published by The Company of Biologists under the terms of the Creative Commons Attribution License (https://creativecommons.org/licenses/by/4.0/).

It remains formally possible that transcriptional de-repression leads to the increased expression of MEX-3 in the germline, although the RNA-seq data shows reduced mRNA abundance despite the increase GFP expression. We have added text to clarify this in the manuscript (p. 18)

The ribosome connection the authors report is intriguing. Elongation rates have been linked to thermogenesis in mammals - PMID: 31417118, PMID: 25353634, PMID: 30890561. In addition to detection of ribosomal proteins, do the authors see any misregulation of these factors? That would be a fascinating parallel.

This is an interesting hypothesis. We looked into the factors responsible for linking elongation rate to thermiogenesis in mammals (AMPK and EEFK) in our data sets. Intriguingly, one of the two AMPK homologs is upregulated in the 3'UTR deletion mutant. The other AMPK homolog and the EEFK homolog are unchanged. We added a description of this on page 21. We plan to look into this further in a future study.

How do differences in mRNA abundance that encode heat shock proteins explain the phenotype with the pseudomonas pathogen? Can the authors speculate on that facet of the story?

Because pathogen exposure causes a strong decrease in the brood size for both WT and mutant animals, we are loathe to speculate that a unifying mechanism may underly the stress response phenotypes observed. However, we do agree that it is important to make a statement to that effect in the manuscript and have included one on page 22.

<u>Second decision letter</u>

MS ID#: dev.204740R1

MS TITLE: The mex-3 3' untranslated region is essential for reproduction during temperature stress

AUTHORS: Hannah E Brown; Haik V Varderesian; Oscar Lam; Sara A Keane; Sean P Ryder
ARTICLE TYPE: Research Article

Dear Dr Ryder,

I am happy to tell you that your manuscript has been accepted for publication in Development, pending our standard publication integrity checks.

<u>Reviewer 1</u>

I thank the authors for their revisions. All of my concerns have been sufficiently addressed.

<u>Reviewer 2</u>

SUMMARY OF THE ADVANCE MADE IN THIS PAPER AND ITS POTENTIAL SIGNIFICANCE TO THE FIELD

MEX-3 is an essential C. elegans RNA-binding protein with a dynamic expression pattern in the germline: MEX-3 is expression is low in the distal gonad, increases as oocytes mature and is subsequently degraded around the activation of the zygotic genome in the early embryo. Previous work from the Ryder group showed that deletion of the GFP::MEX-3 3'UTR (GFP::MEX-3 delta 3'UTR) results in uniform expression in the gonad. GFP::MEX-3(delta 3'UTR) animals were found to be reasonably healthy at 20C, with only a moderate decrease in fecundity (Albarqi 2021).
Brown et al extend this previous work to show that GFP::MEX-3(delta 3'UTR) animals exhibit significant fecundity and embryonic lethality defects at the stressful culture temperature of 25C. GFP::MEX-3(delta 3'UTR) expression levels are reduced relative to wildtype GFP::MEX-3 at 20C or

© 2024. Published by The Company of Biologists under the terms of the Creative Commons Attribution License (https://creativecommons.org/licenses/by/4.0/).

25C. The embryonic patterning defects of GFP::MEX-3(delta 3'UTR) embryos are less severe than mex-3(RNAi) embryos, indicating GFP::MEX-3(delta 3'UTR) embryos retain some MEX-3 function. Transcriptomics analysis identified many hundreds of genes whose expression is altered in GFP::MEX-3(delta 3'UTR) animals. Roughly half of previously identified heat stress responsive genes are expressed at lower levels in GFP::MEX-3(delta 3'UTR) relative to GFP::MEX-3 animals at 20C, which the authors suggest may account for the temperature sensitive fertility defects of these animals at 25C.
It seems likely that the phenotypes described result from decreased expression of GFP::MEX-3(delta 3'UTR), although this was not directly tested. The gene expression changes in GFP::MEX-3(delta 3'UTR) provide an intriguing correlation with the temperature sensitive fertility defects. However, the importance of these gene expression changes are not tested functionally (this would be a difficult experiment). Additionally, how deletion of the MEX-3 3'UTR leads to changes in these genes remains unexplored.

SUGGESTIONS TO AUTHORS

I am satisfied with the author's revisions and am supportive of publication in Development. The work is well-executed and the inclusion of the mex-3(3'UTR delete) in the untagged background addresses my major experimental concern.

<u>Reviewer 3</u>

The revision addressed my points raised in the prior round.

© 2024. Published by The Company of Biologists under the terms of the Creative Commons Attribution License (https://creativecommons.org/licenses/by/4.0/).
